# An Expanded Notch-Delta Model Exhibiting Long-Range Patterning and Incorporating MicroRNA Regulation

**DOI:** 10.1371/journal.pcbi.1003655

**Published:** 2014-06-19

**Authors:** Jerry S. Chen, Abygail M. Gumbayan, Robert W. Zeller, Joseph M. Mahaffy

**Affiliations:** 1 Computational Science Research Center, San Diego State University, San Diego, California, United States of America; 2 Department of Biology, San Diego State University, San Diego, California, United States of America; 3 Department of Mathematics and Statistics, San Diego State University, San Diego, California, United States of America; University of California, Irvine, United States of America

## Abstract

Notch-Delta signaling is a fundamental cell-cell communication mechanism that governs the differentiation of many cell types. Most existing mathematical models of Notch-Delta signaling are based on a feedback loop between Notch and Delta leading to lateral inhibition of neighboring cells. These models result in a checkerboard spatial pattern whereby adjacent cells express opposing levels of Notch and Delta, leading to alternate cell fates. However, a growing body of biological evidence suggests that Notch-Delta signaling produces other patterns that are not checkerboard, and therefore a new model is needed. Here, we present an expanded Notch-Delta model that builds upon previous models, adding a local Notch activity gradient, which affects long-range patterning, and the activity of a regulatory microRNA. This model is motivated by our experiments in the ascidian *Ciona intestinalis* showing that the peripheral sensory neurons, whose specification is in part regulated by the coordinate activity of Notch-Delta signaling and the microRNA miR-124, exhibit a sparse spatial pattern whereby consecutive neurons may be spaced over a dozen cells apart. We perform rigorous stability and bifurcation analyses, and demonstrate that our model is able to accurately explain and reproduce the neuronal pattern in *Ciona*. Using Monte Carlo simulations of our model along with miR-124 transgene over-expression assays, we demonstrate that the activity of miR-124 can be incorporated into the Notch decay rate parameter of our model. Finally, we motivate the general applicability of our model to Notch-Delta signaling in other animals by providing evidence that microRNAs regulate Notch-Delta signaling in analogous cell types in other organisms, and by discussing evidence in other organisms of sparse spatial patterns in tissues where Notch-Delta signaling is active.

## Author Summary

The nervous system of many animals, including the marine invertebrate *Ciona intestinalis* in our study, develops through a cell-to-cell communication mechanism called Notch-Delta signaling. Mathematical models for Notch-Delta signaling have been developed that can explain the development of animal nervous systems with a dense arrangement of neurons. However, there are several cases where the spatial arrangement is much more sparse; we found that the peripheral nervous system of *Ciona* is one such example. Here, we develop an expanded mathematical model that is able to account for this sparser spacing, and furthermore demonstrate that the spacing can be widened or shortened through changing a single parameter that is influenced by the concentration of a regulatory microRNA called miR-124. The underlying differential equations contain only two variables representing the activity levels of Notch and Delta, and are thus general enough to be applicable to a wide variety of physical and biological systems that exhibit a similar sparse patterning.

## Introduction

Differentiation of tissues during early animal development as well as tissue homeostasis during adulthood requires constant communication between cells. One of the most common ways by which cells communicate with each other is through the Notch-Delta signaling pathway [1]–[4]. Notch-Delta signaling is a fundamental cell-to-cell communication mechanism whereby a membrane-bound Delta ligand in one cell binds to a membrane-bound Notch receptor in a neighboring cell, generating a particular downstream response that depends on cellular context [1], [5]. Studies in several animals have shown that Notch expression is both temporally and spatially widespread [2]–[4], [6], [7]. It is not surprising, then, that Notch-Delta signaling is involved in the development and homeostasis of many tissues, most notably those of the nervous system [7], but also within the heart, kidney, liver, pancreas, breast, inner ear, prostate, thyroid, respiratory system, immune system, and many other cell types (reviewed in [1]).

**Figure 1:**
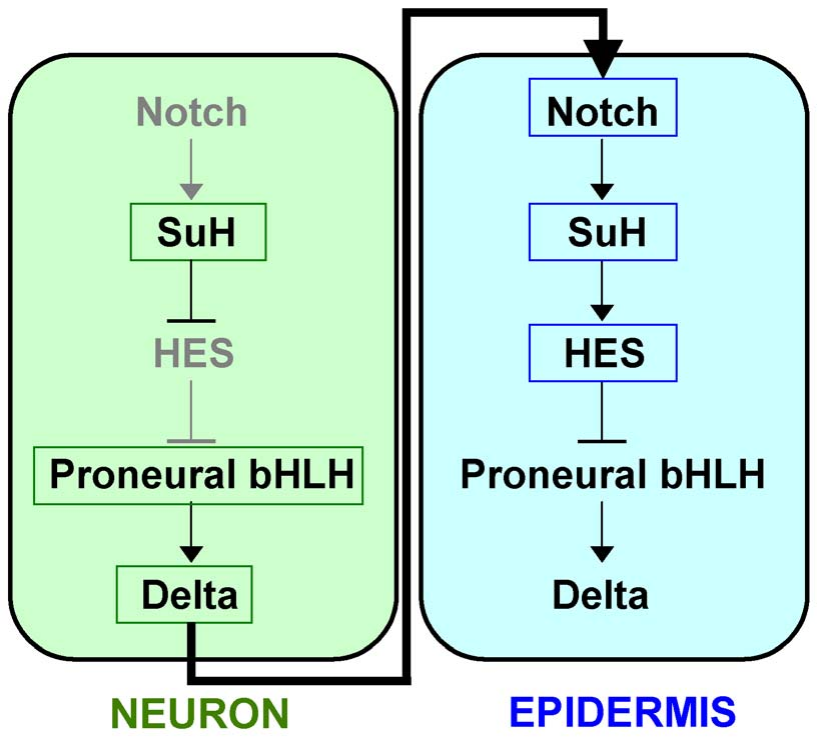
Core Notch-Delta signaling pathway.

Although the specific molecular factors and interactions are remarkably complex and vary among different organisms and cell types, the core Notch signaling pathway is relatively simple and is conserved across all bilaterian animals [1], [3]. The core pathway consists of five main components: a Notch receptor, a CSL family transcription factor (TF), the Hairy and Enhancer-of-split (Hes) family of TFs, the basic helix-loop-helix (bHLH) proneural TFs, and a Delta ligand (Figure 1). In most animals there are multiple genes that encode each component. For example, mammals have four Notch receptor genes and at least seven genes for Hes family members that mediate Notch-Delta signaling in different tissues [8], [9].

Most importantly, experimental studies have shown that neighboring cells, which communicate via Notch-Delta signaling have opposing expression patterns of these five core components [1], [5], [10]. In the signal-sending or Notch-suppressed cell, only the bHLH proneural TFs and Delta are constitutively active, while Notch and Hes expression are suppressed. This suppression is thought to be mediated in part through *cis*-inhibition of Notch by Delta within the same cell [2], [11], [12], and through loss of signaling feedback because Delta is downregulated in the neighboring cell [13], [14]. Conversely in the signal-receiving or Notch-activated cell, Notch and Hes are active, while Delta and bHLH proneural gene expression, even if initially active, are eventually suppressed by a Hes family member [5], [10]. Notch-Delta signaling is often used in a process called lateral inhibition, where the signal-sending cell eventually differentiates into one cell type while inhibiting the signal-receiving cell from adopting the same developmental fate [15]–[17]. Finally, the transcription factor CSL functions as a repressor of Hes family members in the signal-sending cell but becomes an activator of Hes genes in the signal-receiving cell [18], [19]. This functional switch of CSL from repressor to activator occurs when the intracellular domain (ICD) of Notch translocates to the nucleus where it displaces a co-repressor complexed with CSL [2].

With this biological background in hand, several mathematical and computational models have been developed over the years to try and quantitatively explain the dynamics of Notch-Delta signaling [12], [20]–[24]. These Notch-Delta models usually fall into one of two categories: comprehensive models and minimal models. In comprehensive models, all of the experimentally validated (and sometimes solely computationally predicted) molecular components are represented as separate variables, and all of the known or predicted interactions are represented as separate equations in the model [23], [24]. Although complex, these models have led to some key insights into the specific dynamics of particular Notch-Delta pathway genes. For example, one model that incorporated extensive feedback between Notch, CSL, and Hes resolved the long-standing issue that Hes can act both as a bistable switch and as an oscillator by showing that the transition between these two states can occur by tuning a single parameter, the Hes1 repression constant [23]. Another model incorporating Goodwin-modified biochemical kinetic equations for transcription, nuclear export, translation, and DNA-binding and dimerization of each factor showed the importance of the decay rate of Hes1 [24]. However, one drawback of comprehensive models is that they are usually based on experimental data from one particular cell type and, therefore, are not generalizable to other systems.

By contrast, in minimal models only the core molecular components and interactions, which capture the overall, essential Notch-Delta signaling dynamics, are represented in the differential equations. Unlike comprehensive models, minimal models have the advantage of being applicable to many biological contexts and are also more amenable to parameter sensitivity and stability analyses, which can shed important insight into the dynamics of the system. The first minimal Notch-Delta model was published by Monk and colleagues [20], which at its core is a simple two-cell model with a feedback loop involving just two variables: Notch and Delta. Because the core cascade is essentially linear, they postulated that the Notch variable could represent the quantity of activated Notch protein (*i.e.*, Notch ICD) in the cell or the quantity of downstream Hes TF [20]. The production functions representing Notch-Delta interactions could be modeled using Hill functions, which are commonly used to model protein-protein as well as protein-DNA interactions [12], [20], [25] and for which we now have extensive experimental confirmation through biochemical studies [12], [26]. Through their model, Monk and colleagues demonstrated that such a feedback model results in a checkerboard spatial expression pattern of Notch and Delta, which mimics the Notch-Delta pattern found in several biological contexts for which lateral inhibition occurs [20], [21], [27]. With lower cooperativity (*i.e.*, a lower Hill coefficient), occasionally a spacing of two or three cells can occur [20]. Subsequent models over the next several years were for the most part variations of the original Monk model (*e.g.*, [21], [22]). Eventually, growing experimental evidence of *cis*-inhibition of Notch by Delta led to an updated model by Elowitz and colleagues that incorporated this interaction [12]. Such *cis*-inhibition was thought to facilitate Notch-Delta lateral inhibition, and indeed the expanded model resulted in faster dynamics, sharper checkerboard patterning and greater robustness to noise [12].

While the Monk and Elowitz models can explain the patterning in some biological systems such as ciliated cells in the early Xenopus ectoderm [21], there are cases in both invertebrates [28]–[34] and vertebrates [7], [35]–[37], where Notch-Delta signaling is clearly active but the pattern is not checkerboard. In many cases, the pattern is much more random and sparse, where the spacing between signal-sending cells can range from a single cell to dozens of cells in between [30], [31], [33]. For example, studies in zebrafish and chick neuroepithelial tissues have demonstrated a gradient of expression for Notch and/or Delta [7], [36], [37]. Also, the sensory organ precursor (SOP) cells of the *Drosophila* thorax that give rise to microchaetes are spaced about five cells apart when fully developed [5], [28]–[30], [38]. A pair of studies demonstrated that SOPs in wild-type *Drosophila* extend dynamic projections called filopodia, and that these filopodia express graded amounts of Delta along the filopoidia and allow the SOPs to reach out and activate Notch signaling in non-neighboring cells [30], [31]. Another form of extended communcation in Notch signaling can occur through a process called lateral induction, in which a Delta-bound Notch receptor in the signal-receiving cell can induce the expression of other ligands, which signal Notch in downstream cells [39]–[41]. Several authors analyzed more generalized models[42]–[44] with nearest neighbor or juxtacrine inhibition and induction and found these systems could generate Turing solutions[45] from a homogeneous steady-state with various wavelengths. Thus, a model for a juxtacrine system can produce stable periodic patterns with larger spacing between peaks of Delta activity. Hence, in addition to neighboring-cell lateral inhibition, a form of communication leading to long-range patterning can also operate in the context of Notch-Delta signaling. Since these filopodia are wide at the base but gradually thin out towards the tip, this suggests a concentration gradient where cells touching near the base of filopodia receive stronger Notch activation compared to cells in contact with the tips.

In this report, we present a minimal Notch-Delta model, which expands upon the previous Monk and Elowitz models [12], [20] by adding a simple nearest-neighbor Notch gradient term that makes it possible for the system to exhibit long-range effects on cell morphogenesis. We show that incorporation of a Notch activity gradient term is able to produce a sparse pattern of Delta expression whereby Delta-expressing cells can be spaced many cells apart. In our studies, we focus on the patterning of larval tail epidermal sensory neurons (ESNs) within the peripheral nervous system (PNS) of the ascidian *Ciona intestinalis*. We quantify the number and spacing of ESNs in wild-type larvae, and show that our expanded Notch-Delta model accurately reproduces the experimentally observed ESN pattern [33], [34], [46]. Ascidians are invertebrate chordates and are the closest invertebrate relatives of vertebrates [47]. As such, they occupy an important phylogenetic position for understanding how molecular developmental pathways evolved when invertebrates and vertebrates diverged from their last common ancestor [34], [48]. Sensory neurons, like those in the *Ciona intestinalis* PNS, the mechanosensory bristles found in *Drosophila*, and the hair cells of the mammalian inner ear, are thought to have evolved from a common ciliated sensory-neuron precursor [34], [49]. Since Notch-Delta regulated tissues in flies, ascidians, zebrafish, chick and mice have all been shown to exhibit sparse spatial patterning [7], [30], [31], [36], [37], our model suggests that Notch-Delta-mediated long-range inhibition may be broadly conserved in bilaterians.

We also demonstrate that regulation of Notch-Delta signaling by microRNAs (miRNAs) is conserved across bilaterians. The miRNAs are a class of conserved small RNAs that regulate expression of target genes through transcript destabilization, deanylation and/or translational inhibition, leading to downregulation of the protein product [33], [50]. Previously we demonstrated that in *Ciona* the miRNA miR-124 downregulates Notch and all three Hes factors, and that these operate in a negative feedback loop [33]. Here, we show that miRNA-mediated regulation of Notch signaling can be incorporated into the parameter representing the decay rate of the Notch variable, and that modulation of the Notch decay rate in the model accurately mimics the ESN pattern observed in wild type larva and in miR-124 overexpressing transgenic larvae that have altered ESN spacing patterns. Finally, through a bioinformatics analysis we demonstrate that the majority of miRNAs expressed in sensory cell types of other animals are predicted to target Notch pathway genes in their representative systems, suggesting that miRNA interactions with the Notch signaling pathway may be functionally conserved.

## Results

### Sensory neuron patterning in *Ciona intestinalis* is sparse and irregular

In *Ciona intestinalis*, the tail epidermal sensory neurons (ESNs) differentiate from epidermal precursor cells within the dorsal and ventral midlines. Previous work in our lab and others [33], [34], [46], [51] has qualitatively shown that the midline ESN pattern is very irregular, although a quantitative investigation of the number, spacing and distribution of ESNs has not been done. Thus, we began by quantifying ESN numbers and ESN spacings in wild-type embryos by immunohistochemically-labeling the associated cilia with an anti-acetylated tubulin antibody. We focused on an older developmental stage (22 hours post-fertilization at 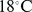), when the larvae have extended their tails and when the final midline ESN pattern has emerged [32]–[34]. To identify the midlines, we generated transgenic embryos expressing either an Acete-Scute homolog(ASH) RFP reporter or a Delta RFP reporter (see Materials and Methods) [34]. To identify the ESNs, we used fluorescent microscopy to image cilia in embryos immunohistochemically detected with an antibody against acetylated-tubulin. ESN cell nuclei are smaller than those found in the surrounding epidermal cells, and could be visualized with DAPI staining [32].

**Figure 2:**
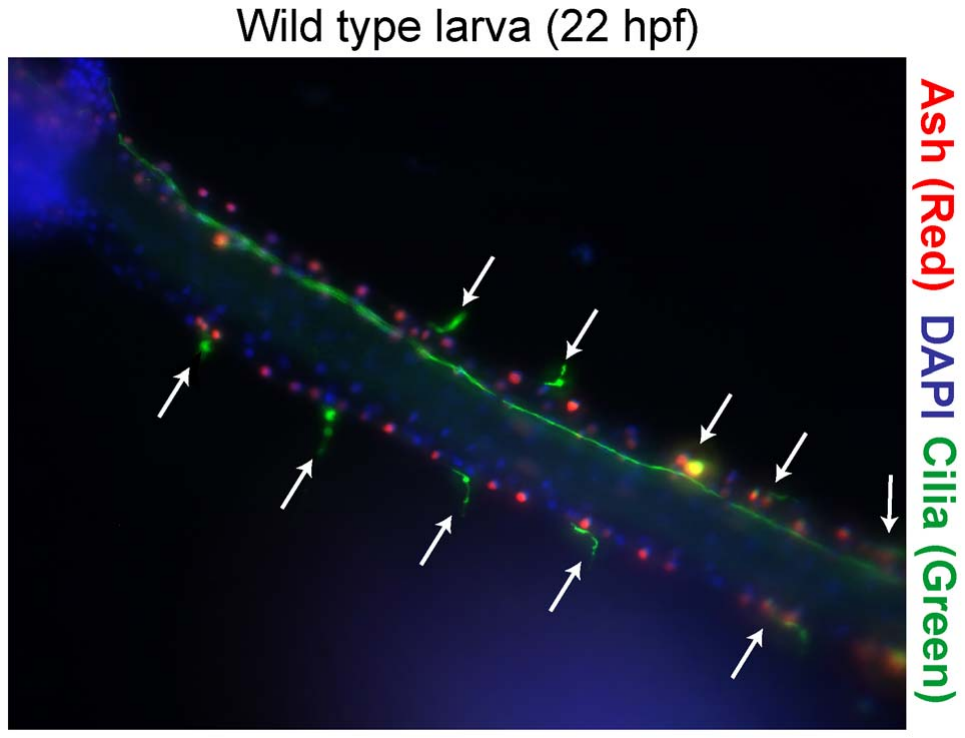
Wild-type sensory neuron pattern in the *Ciona* larval PNS. A representative transgenic embryo expressing an ASH::RFP reporter in midline cells. Cilia (green) have been detected with an anti-acetylated tubulin antibody; ESN cilia (arrows). Coupled with DAPI staining (blue), these markers facilitated counting the number of ESNs and the number of midline cells between ESNs.

**Figure 3:**
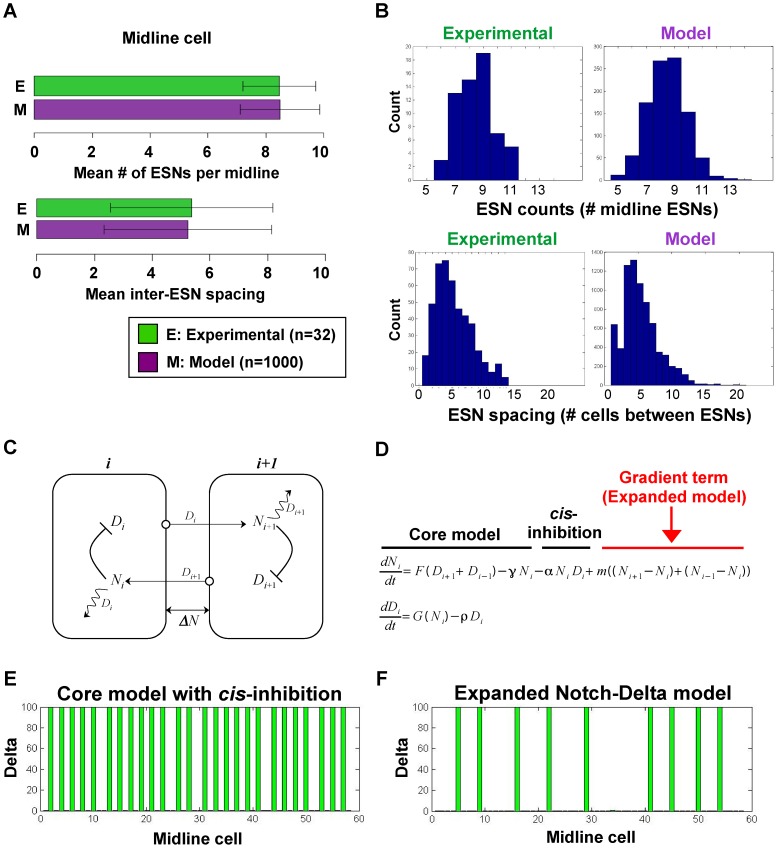
Expanded Notch-Delta model. (A) Monte Carlo simulations show that our expanded model produces ESN numbers and spacings that match with experimentally determined values. (B) The distributions for the number and spacing of ESNs, including the minimum/maximum and variances of the distributions, are all very similar between model and experiment. For the top graphs, the y-axis shows the number of midlines with the given number of ESNs. For the bottom graphs, the y-axis shows the number of times a given ESN spacing occurs. (C) Schematic showing the intra- and inter-cellular interactions between Notch and Delta. The squiggle arrow represents *cis*-inhibition of Notch by Delta. Note that for clarity only two cells are shown, but the interactions extend over a linear array of cells. (D) The general form of the ordinary differential equations of our expanded model for Cell 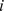, with addition of a Notch activity gradient term indicated in red. (E–F) Shown are the equilibrium values of Delta after a typical run of our expanded model in comparison with the original core model [12], [20].

Figure 2 shows a representative embryo used for quantitation. In agreement with previous qualitative observations [33], [34], [51], we found that the number, distribution, and spacing of ESNs varied considerably from embryo to embryo (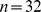 embryos quantitated across three independent biological replicates). Overall, we found no obvious differences between the number of midline cells, number of ESNs or the spacing between ESNs along the dorsal versus ventral midline at 22 hours post-fertilization (see Figure S1). Therefore, we only considered statistical averages per midline without distinction between dorsal and ventral counts. No larvae had fewer than six ESNs per midline, consistent with previous observations that six dorsal midline precursor cells express Delta early in embryogenesis prior to midline formation [32]. We observed as many as eleven ESNs along a single midline in 22 hr larvae. We never observed more than eight or nine ESNs in earlier embryos (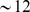 hours post-fertilization) [34], suggesting that ESNs continue to be specified as the larval midline develops. We observed a variable pattern in ESN spacing with as few as one and as many as thirteen epidermal (non-ESN) cells separating consecutive ESNs. We never observed two ESNs next to each other, consistent with the hypothesis that Notch-Delta-mediated lateral inhibition is active between neighboring ESN-epidermal cells [32], [33]. These results are summarized in Figure 3A–B. Regarding the distribution of ESNs, we found no apparent bias of ESN position along the anterior/posterior axis. However, we did observe that consecutive ESNs spaced at least ten cells apart were almost invariably flanked on at least one side by two or three ESNs spaced very closely (Figure S2).

### An expanded Notch-Delta model exhibiting long-range ESN patterning

With this quantitative experimental data in hand, we began drafting a Notch-Delta mathematical model that could adequately explain the patterning of midline ESNs in *Ciona*. We began with a linear array of 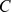 cells representing a single midline at a fixed time point. As mentioned, we did not notice any obvious differences between the dorsal and ventral midlines at the larval stage (see Figure S1), so our model is appropriate for modeling either midline. Future models will modify this static array into a dynamic array that includes cell division. This 1-D model could also be easily expanded to a 2-D array for modeling planar systems such as the proneural clusters in *Drosophila* [5], [12], [20], [30].

Consistent with previous minimal models, each cell tracks the activity of just two biochemical species, Delta (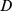) and Notch (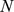) or some closely affiliated biochemical species, such as a transcription factor directly linked to these primary proteins. Note that because our model can be applied to other biochemical and physical systems, when we present the differential equations of our model below, we will denote the Delta and Notch species more generally as 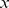 and 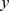, respectively. As discussed in the original Monk model [20], 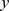 could be taken to represent the quantity of activated Notch (*i.e.*, Notch ICD) in the cell; or it could be taken to stand for the quantity of downstream Hes TF in the cell. In addition, since the Notch-SuH-Hes cascade is linear and exhibits bistability (*i.e.*, there are only one of two stable states for each node - either all "ON'' or all "OFF''), we can regard the states of Notch, SuH and Hes as equivalent, and can therefore consider any of these or all of these lumped together as the variable 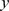 [52]. Analogously, since we know that the bHLH proneural genes are expressed in a linear cascade and are upstream of Delta [34], 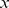 could represent the quantity of membrane-bound Delta in the cell or could incorporate the activity of the upstream proneural TFs [52].

Figure 3C shows a schematic of our model for the interaction between neighboring cells. All the cells in the linear array interact with their nearest neighbors with the exception of the end cells. The model localizes 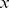 inside the cell or expressed on the cell surface to signal only the neighboring cells. It is repressed internally by 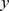 and activates neighboring cells to stimulate production of 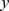. The species 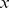 also catalyzes the *cis*-inhibition of 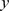 inside the same cell. The production of 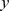 depends on the activity of 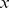 in the neighboring cells. Both species have linear decay terms based on the half-lives of Notch, 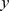, and Delta, 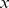. Finally, we include a communication term for 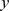 to neighboring cells based on the gradient in activity of active Notch or a related biochemical species between the cells. The addition of this gradient term is the primary distinction of our model from previous Notch-Delta models. In earlier models, interactions are exclusively with neighboring cells, which restricts the patterning to primarily alternating on and off states, while our model by including a Notch activity gradient can simulate larger cell spacings, which match that found in *Ciona* and in other analogous Notch-Delta systems [7], [30], [37]. Although the exact mechanism of long-range communication is currently unknown in *Ciona*, we favor a nearest-neighbor Notch gradient term versus other possibilities based on our current biological knowledge of Notch-Delta signaling in the *Ciona* PNS (see Discussion).

All of the above interactions represent the core conserved interactions of Notch-Delta signaling and are supported by extensive experimental evidence [4], [5], [10], [53]. Let 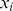 and 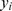 be the activity levels of Delta and Notch in cell 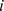, respectively, then the dynamics for the model described above is given by the following system of differential equations: 
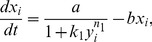
(1)

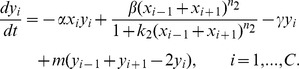



In the system above, we let the boundaries satisfy: 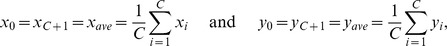
where 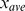 and 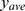 are the average activity levels of Delta and Notch over the entire array of cells. Clearly alternate boundary conditions could be considered, although other common boundary conditions such as zero or periodic boundary conditions are not appropriate for modeling the *Ciona* midline.

The functions and the parameters in the model are common in biochemical control models [12], [20], [25], [54]. The essential form of each function is the same as those found for earlier minimal Notch-Delta models [12], [20] (Figure 3D). A full explanation of each of these functions and parameters can be found in Materials and Methods, but here we briefly mention the functions and parameters that are immediately relevant for our analysis. The first term on the RHS of the 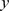 equation represents *cis*-inhibition by 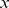. The parameters 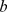 and 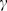 are the linear decay rates of Delta and Notch or a related biochemical species, respectively. Because our biochemical species do not distinguish between mRNA and protein levels, we may take them as representing mRNA and/or protein decay rates. The last term in the 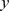 equation is the linear gradient term representing long-range communication. This cell-to-cell gradient term could result from bound Notch molecules self-signaling to create a gradient-like pattern of activity. It could be the result of another signaling biochemical closely aligned with Notch, but not necessarily bound so strongly to the membrane. From a modeling perspective this gradient form of nearest neighbor communication is the simplest mechanism of long-range patterning and makes a good first order approximation to the kinetic interactions of this signaling pathway. For the remainder of the article, we will refer to 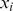 as 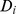 and 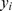 as 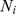 to associate the model state variables with the Delta (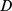) and Notch (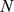) pathways.

### Monte Carlo simulation of expanded model reproduces sensory neuron pattern in *Ciona*

We wrote programs to simulate our Notch-Delta model using the Matlab 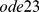 solver. We began our simulations with random low activity levels of 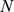 and 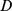 in all cells and first observed the qualitative behavior of our system over time. After some time passed, a few cells developed a high level of 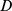. The high level of 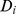 in Cell 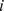 suppressed 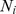 in the same cell (*cis*-inhibition) and led to above average levels of 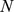 in Cells 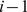 and 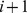 (lateral inhibition). Via the linear gradient term, subsequent neighboring cells had decreasing levels of 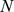, until some critical threshold was reached with 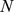 sufficiently low that another cell could once again produce a high level of 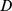, then the pattern repeated. The dynamical system exhibited very stable behavior for the levels of 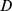 and 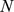 in the immediate region near Cell 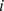. However, we observed decreasing stability of the activity levels as levels of 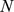 decrease.

When there was sufficient spacing between cells with high levels of 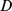, then we observed later development of cells with high levels of 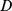 in the intervening area of cells. These later developing cells arose from two distinct dynamical behaviors. In one case there were sufficiently low levels of 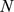 far from the ones with high levels of 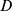, resulting in the smooth development of an intervening cell with a high level of 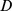. This case was most common early in the simulation. In the second case, the levels of 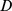 and 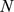 oscillated in the regions between stable areas of high 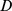, with the amplitude of the oscillations appearing to increase with increased ESN spacing. With enough spacing, the oscillations increased until a threshold was crossed, allowing the development of another cell with a high level of 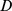.

Because of the random initial conditions, different patterns of cells with high 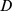 levels arose. The spacings in these patterns depended strongly on the parameter values; however, after sufficient time a stable pattern emerged. A representative example is shown in Figure 3F. Note that spacings of more than two cells cannot be achieved with either the original Monk model [20] nor the model incorporating *cis*-inhibition [12] (Figure 3E).

To determine if our model could explain the ESN pattern along the *Ciona* midline, we ran a Monte Carlo simulation with 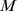 = 1000 runs over 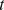 = 4000 time steps for each run, and compared the number, spacing, and distribution of high Delta-expressing cells with that of the ESNs from wild-type embryos. Our simulations used the parameter values listed in Table 1.

**Table 1:** Parameters used for the Monte Carlo simulations.

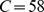	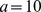	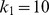	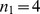	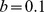	
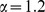	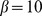	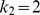	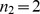	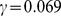	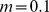

The parameters were chosen for the following properties. The value for 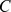, the number of cells, was chosen to match the average number of midline cells from our experiments. The parameters 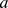, 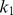, 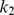, 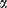, and 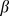 were fairly arbitrary, although they were chosen based on our knowledge of similar biochemical control models from previous work [12], [20], [25], [54]. As off-diagonal elements, these parameters should not be as significant to the behavior of the system as the other parameters (though the 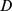-mediated decay 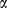 could be an important parameter when considering the effect of modulating *cis*-inhibition, as in a previous study [12]). The most significant parameters for the switching behavior are the parameters 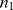 and 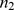, the Hill coefficients. These are chosen be be greater than one, but not too large to be biologically relevant. The decay rates 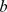 and 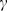 along with the gradient parameter 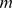 are very significant as we will see in the bifurcation analysis. In particular, 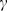 will be important when we consider the effect of microRNA-mediated regulation of Notch signaling. For these simulations, 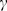 was adjusted so that the average number of high-Delta cells over the 1000 runs closely matched the number of ESNs from wild-type experiments. Since Delta is an epidermal sensory neuron marker [34], throughout the text we will refer to high-Delta cells and ESNs interchangeably.

Figure 3F shows the end results of a typical run, with Movies S1 and S2 showing the dynamics of two separate runs starting with random low initial conditions for both Delta and Notch. Both movies show the appearance of new ESNs in regions where the spacing between existing ESNs is large. In movie S1, the levels of Notch and Delta settle into a very stable equilibrium; while in movie S2, the levels of Notch in the cells between the ESNs at Cells 27 and 39 show distinct stable oscillations. Figure 3A–B shows the statistics for the number and distribution of ESNs and inter-ESN spacing from 1000 runs. While agreement between the average number of ESNs predicted by the model and experimentally observed in larvae is expected, surprisingly the distribution of ESNs and the average ESN spacing matched very well with experimental observations. The majority of runs in our Monte Carlo simulations produced between 6 and 11 ESNs, with a peak of 9 ESNs, matching experimental observations. There were some instances of outliers on either side in our simulations, although if we were able to quantify an equivalent number of embryos (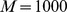), we might expect some experimental outliers as well. Similarly, the ESN spacing in our simulations matched experimental observations, with the frequency histograms following an identical gamma distribution with a peak at 4 cells and dropping off after 13 cells. There were a few rare outliers where ESN spacing exceeded 13 cells. When we analyzed these outliers more closely, we noticed that these large spacings were flanked on at least one side by two closely ESNs (Figure S2). These closely spaced ESNs likely stabilize the cells within the large-spacing valley. This is in agreement with our experiments showing that cases of high inter-ESN spacing were flanked on at least one side by consecutive ESNs with tight spacing (Figure S2). Finally, we note that our model has a disproportionate number of one-cell spacings compared with experimental observations. This is likely due to the intense stability of the high-Delta cells and the strong effect of lateral inhibition in our model.

We chose our Hill coefficients 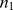 and 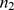 based on our knowledge of previous biochemical control models [12], [20], which produced the reasonable fits seen in Figure 3A–B. However, we know that changing the coefficients, 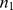 and 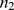, affects the lateral inhibition and induction of immediately neighboring cells and results in differing distributions of cell spacing. Simulations with 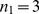 and 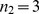 produced significantly broader distributions (similar means, but a much larger variance), while 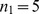 and 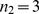 produced a much narrower distribution (similar mean with a smaller variance). Our modeling experiments suggest that increases, especially in 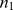, would produce more two-cell spacings at the expense of one-cell spacings as suggested in the experiments. However, since Figure 3A–B shows our model adequately represents the experiments, we chose to center our studies around the case 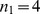 and 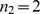.

### Stability analysis explains midline ESN patterning

A stability analysis is used to determine equilibrium states of a system and the change in behavior of a system as the parameter values vary. This analysis is important because it allows us to determine the possible ESN patterns that can be produced from our model, and to rigorously determine if our model can really explain the biology. We therefore designed programs to help numerically find equilibria and allow the stability analysis of the equilibria. The stability analysis uses the Jacobian matrix analytically derived from linearizing the system (1) (see Materials and Methods).

There is a unique homogeneous equilibrium for system (1). Related systems [20], [42]–[44] have been analyzed in terms of the stability of the homogeneous equilibrium, showing the existence of Turing solutions. For system (1) with the parameters in Table 1, there is a homogeneous equilibrium with 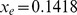 and 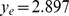, which is unstable with multiple positive eigenvalues. Since the experimental studies do not suggest a periodic pattern, we did not explore Turing solutions. Our primary interest was the behavior of the many inhomogeneous equilibria.

The Monte Carlo simulations showed the variety and large number of possible stable equilibria for model (1). This model can easily reproduce the stable alternating pattern of the previous Monk [20] and Elowitz [12] models. These models are very similar to (1) with 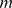 = 0 and 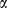 = 0, respectively; however, non-zero values of 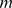 and 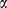 allow the richer stable patterns shown in the Monte Carlo simulations. From the many equilibria for this system we chose to systematically explore the stability of the system with different spacings of high 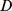 levels. The numerical observations showed decreased stability of the cells some distance from the cells with high 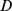 levels, so we wanted to explore the nature of any bifurcations leading to limits on the spacing of the cells. Below we present the stability analysis for different ESN spacings, giving information about the dominant eigenvalues and commenting more about the observed eigenvalue structure. The parameters we use in this analysis come from Table 1. In biological terms, the eigenvalues and eigenvectors tell us the differentiation state of each of the midline cells. Roughly speaking, if a cell aligns with an eigenvector associated with the most negative eigenvalues, then it is stable and has fully differentiated into an ESN. The cells that align with the largest components of the eigenvectors associated with eigenvalues with positive real part are unstable and remain bipotent.

To help minimize the effects of the boundary, we varied the number of cells in our simulations to be as close as possible to 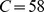 (which is the average number of midline cells found in all of our experiments), while maintaining symmetry at the boundaries. Suppose two consecutive ESNs are Cell 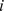 and Cell 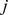, then define 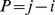 (1 ESN and 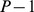 epidermal cells). We numerically find the equilibrium of (1) for each value of 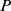. From the linearized form computed in Materials and Methods, we can readily find the eigenvalues and eigenvectors for this system. Table 2 summarizes the results of different spacings using the parameters from Table 1 and shows the dominant eigenvalues of the system.

**Table 2:** Different spacings of high 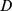 given by 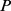.

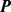	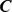	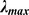	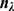	Stability
5	58	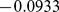	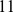	Stable
6	59	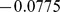	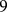	Stable
7	54	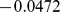	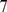	Stable
8	55	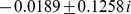	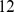	Stable
9	61	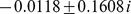	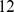	Stable
10	59	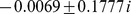	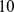	Stable
11	53	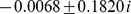	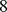	Stable
12	59	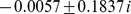	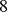	Stable
13	63	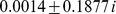	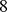	Unstable

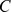
 gives the number of cells in the array. 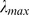 gives the dominant eigenvalue, and 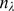 gives the multiplicity of the dominant eigenvalue.

The linear stability analysis of (1) with the parameters from Table 1 and the spacings and numbers of cells from Table 2 gives a better understanding of this system. The overall stability of system (1) is determined by the real part of the dominant eigenvalue, 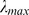, with this system being asymptotically stable if and only if 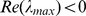. However, this is a high-dimensional system, and different components of the model behave differently near an equilibrium based on its structure. The time-series local behavior of different components vary more or less depending on their location, and their fate can be understood by careful examination of the eigenvector associated with specific eigenvalues.

With MatLab we computed all eigenvalues and eigenvectors for each of the cases in Table 2. In every case we had the smallest eigenvalue 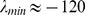 with a multiplicity matching the number of cells with high levels of 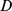. By examining the corresponding eigenvectors, we found the largest components centered on the highest 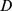 (lowest 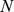) values. (Note that because of the scaling, the 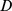 components of the eigenvectors are much smaller than the 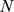 components, so we compared only relative size within 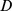 or 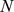 components.) Each of the eigenvectors associated with one of the eigenvalues, 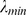, had a large 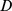 component and a large 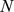 component at one of the ESN positions with all other components at least four magnitudes of order smaller. This agrees with our observation that the model produces extremely stable regions near cells with high levels of 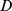, *i.e.*, differentiated ESNs.

The real part of the dominant eigenvalue, 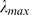, becomes larger as the spacing, 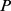, increases. This correlates to the decreasing stability of the levels of 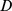 and 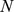 as the spacing increases. The multiplicity of 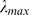 matches the number of interspacings between cells with high 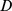. When examining the particular components of the corresponding eigenvectors, the patterns were more complex, spreading across several interspacings. However, the maximum 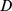-component occurred near the center of the interspacings with the maximum 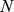-components flanking either side of the maximum 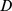. This is in line with the observation that the next highest 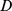-component always occurs near the middle of our cells with high levels of 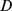, while the flanking cells show the highest 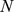 responses in agreement with Notch being highest in cells neighboring a cell with high Delta.

**Figure 4:**
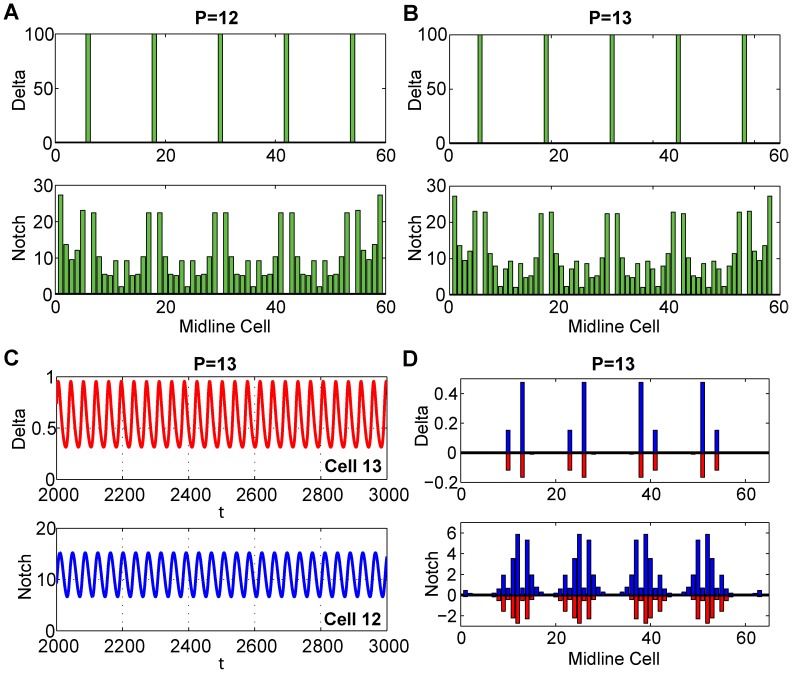
Stability analysis of the ESN spacing, *P*. (A–B) The top graphs show the equilibrium values for the Delta and Notch levels. (C–D) The bottom graphs examine the unstable case 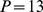 and show the time varying oscillations (left) of Cell 13 for 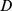 and Cell 12 for 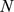 and the variation from the equilibrium for all cells (right).

As 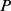 increases, the real part of 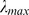 changes signs between 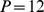 and 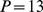, giving a Hopf bifurcation. Figure 4A–B shows the equilibrium state of the system at 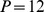 and 13, and the simulations show distinct oscillations. From Table 2, any simulation with 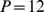 would show damped oscillations with the solution settling to the equilibrium. The eigenvalue for 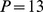 has a frequency of 0.1877, which implies a period, 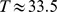. Figure 4C–D shows the oscillatory solutions from a simulation with 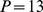, and the period of oscillation agrees with the frequency of 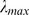. The eigenvectors of 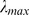 with 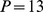 show a structure very similar to the graph in Figure 4D, where variation for each cell from its equilibrium is displayed. The variation in 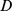 is very small (about 1%) compared to the size of the high Delta cells, while the oscillations in 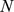 are quite substantial relative to the equilibrium Notch levels, especially in the cells flanking the cell, which has the greatest variation in 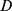 near the middle of the interspacing region. This example with 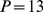 has an unstable equilibrium, but its oscillations are insufficient in magnitude to cross a threshold and pass to a different equilibrium with high Delta cells between the ones shown in Figure 4B. We note that slightly different initial conditions away from the equilibrium will cause new ESNs to arise, indicating that the basin of attraction for the 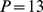 equilibrium shown is quite small.

Once 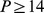, our numerical algorithms cannot find an equilibrium solution to linearize around and any simulation results in new ESNs appearing, indicating the 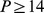 spacings are too unstable when evenly spaced. Thus, our model suggests that when the number of cells between ESNs becomes too large, then new ESNs appear in between. Importantly, in agreement with this bifurcation analysis on 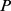, our wild type experiments show a maximum spacing of 13 cells between ESNs. This suggests that if the midline cells divide and the spacing becomes greater than 13, the instability of such a state will cause a new ESN to appear. Also recall that with our parameter values the spacing mean and distribution matched the wild type experiments. Thus, our experimental results are in harmony with our numerical analysis of the spacing, 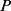.

The analysis above examines discrete changes in the spacing, 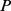. We next chose to explore continuous changes with the gradient parameter 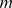. For these studies we set 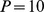, 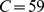, and all other parameters from Table 1 except for 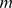. From the analysis above we know that instabilities should cause an ESN to appear midway between and create a 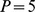 pattern. Our interest is to determine something about the dynamics of change from a larger spacing, 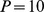, to a smaller spacing, 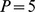.

**Figure 5:**
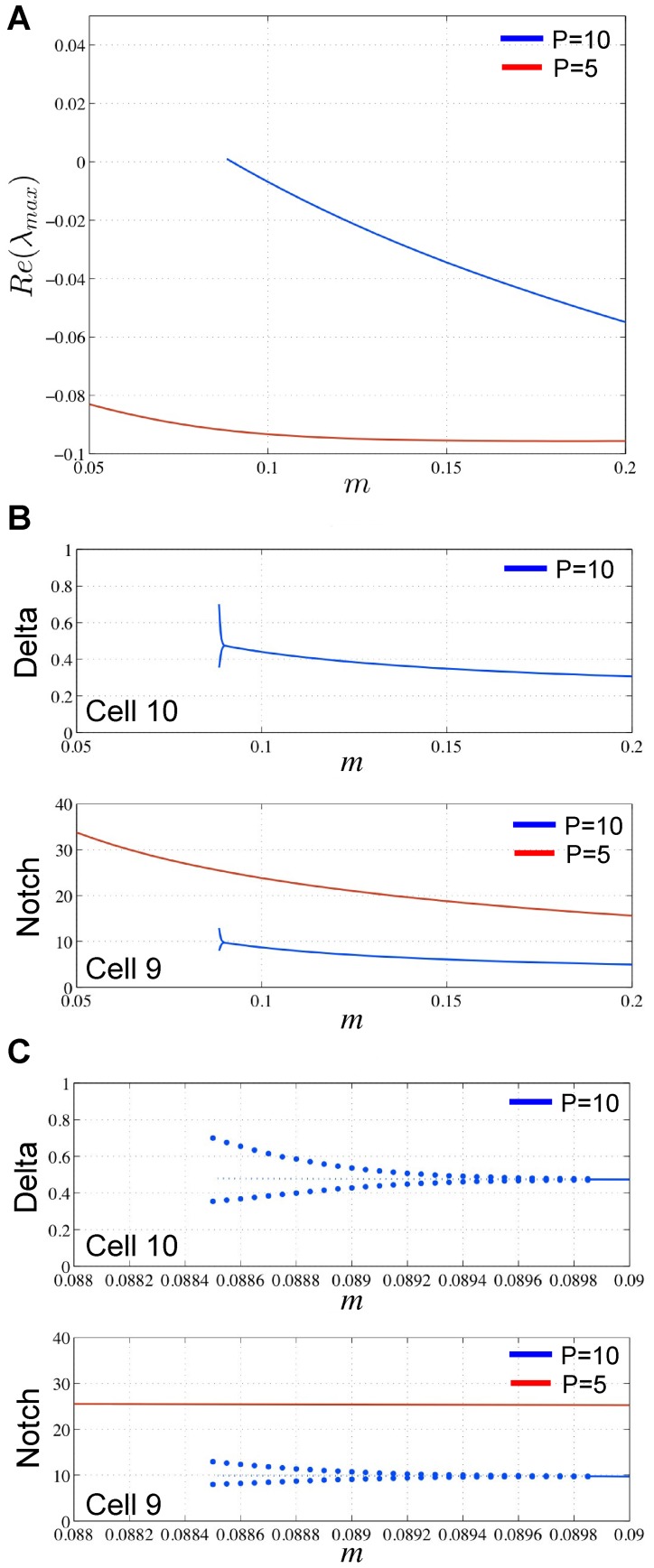
Stability analysis of the parameter *m*. (A) 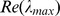 for 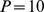 (blue) and 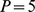 as 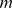 varies. (B) The equilibrium levels of Delta (top) and Notch (bottom) for our cases 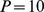 (blue) and 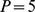 as 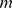 varies. (C) Extreme close-up of the graph in (B), showing the supercritical Hopf bifurcation with the stable oscillating periodic orbit.

Decreasing 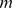 in essence shortens the effective distance of Notch signaling. As noted before, when 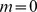, the Monk model only produces an alternating pattern of high 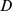 and 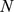 with no spacings larger than two and most being one. Thus, we expect the stability of the 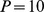 pattern to be lost as 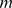 decreases. We studied the linear stability of the 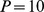 pattern as 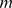 ranged from 0.2 to 0.08845. At the ESNs, where 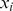 is high, 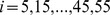, the smallest eigenvalue is 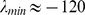, making this region of the cellular array extremely stable. The maximum eigenvalue, 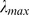 has its eigenvector centered between the cells with high 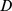. Figure 5A shows the variation in the real part of 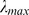 as 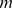 varies. When we decrease 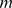 to 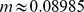, there is a Hopf bifurcation (verified with Auto in XPPAUT), introducing oscillations in cellular activity levels, 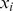 and 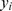. The maximal oscillations in 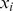 occur in the middle cells, 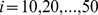, while the maximal oscillations in 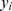 occur in the adjacent cells, *e.g.*, Cells 9 and 11. Figure 5B–C shows the equilibrium levels for 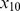 and 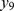, and the maximum and minimum of the oscillating levels after the Hopf bifurcation. As is typical of a Hopf bifurcation, these oscillations increase in amplitude away from the Hopf point.

As 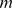 decreases further to approximately 0.0885, the instabilities are sufficient that the solution leaves the basin of attraction for the 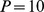 equilibrium. The result is that the solution converges to the very stable pattern where 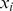 is high at 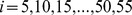, resembling the 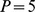 equilibrium. The maximum eigenvalue for this solution is 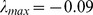, producing a very stable equilibrium. We note that the basin of attraction for this solution is significantly larger than the basin of attraction for the 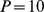 case. Figure 5B–C shows the increase of both 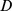 and 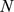 as 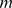 decreases. It appears as though some threshold is reached, which results in 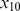 approaching 100 and 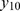 going to very low levels quickly. It is not clear if this transition is smooth and very rapid or if some saddle node bifurcation is occurring. At this time the specific type of bifurcation moving from the 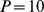 to the 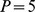 spacing has not been determined and needs further analysis.

**Figure 6:**
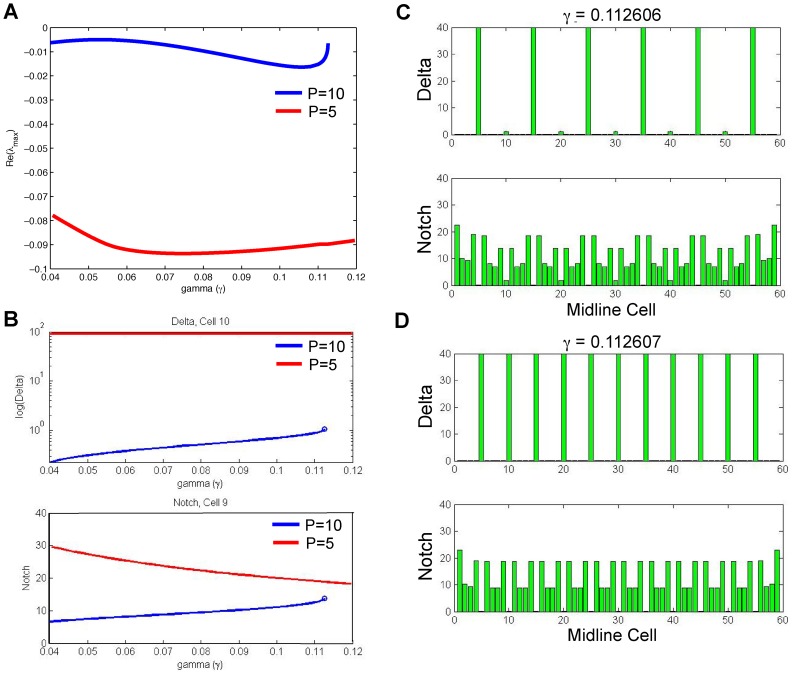
Stability analysis of the parameter 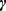. (A) 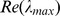 for 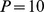 (blue) and 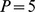 (red) as 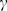 varies. (B) Equilibrium levels of Delta (top) and Notch (bottom) for our cases 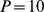 (blue) and 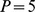 (red) as 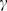 varies. Note that Delta is shown as a semilog-y plot to show the change in Delta. (C–D) Equilibrium levels for Delta and Notch across all midline cells as we cross 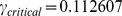.

Finally, we analyzed the change in behavior of the system as we increased the Notch decay rate parameter, 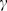. We began with a constant spacing of 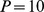 cells and the corresponding value of 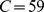 from Table 2, with all other parameters from Table 1. Starting with a low value of 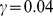, we increased the value of 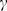 with a step size initially of 0.01. As we stepped from 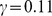 to 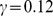, a significant change in the system occurred whereby new ESNs appeared halfway between existing ESNs, similar to what occurs when we decrease 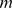. Through repeating this stepping process with decreasing step sizes, we determined the exact value of this critical value of 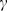 to be 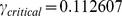. With every iteration of this process, we kept track of the minimum and maximum eigenvalues and associated eigenvectors (Figure 6A), as well as the equilibrium values of 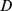 and 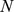 (Figure 6B).

As in the case of 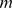, analysis of the min/max eigenvalues and associated eigenvectors revealed that the existing ESNs (*e.g.*, Cell 5) are highly stable, while the middle intervening cells (*e.g.* Cell 10) are in regions of lower stability. However, unlike with 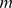, the levels of 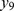 and 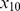 do not exhibit oscillations as we approach the critical value 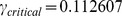 (Figure 6B). The real part of the maximum eigenvalue 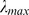 remains negative as we vary 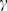, indicating that there is no Hopf bifurcation (Figure 6A). At 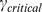, the system moves out of the basin of attraction for 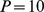 and converges to a new stable pattern with smaller spacings resembling the 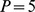 equilibrium (Figure 6C–D). The behavior in Figure 6B is similar to a saddle node bifurcation, but a more detailed analysis is required. As we decrease 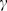 back to 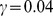, the system remains in the new equilibrium, indicating that this equilibrium is very stable and has a very large basin of attraction. In biological terms, we may interpret this hysteresis effect as the newly formed neurons have committed to their new state and will not easily revert back to being bipotent.

Significantly, our analysis shows that increasing 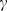 beyond a critical value can produce new cells with high levels of 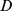, which demonstrates that, based on our model, increasing the Notch decay rate can produce new ESNs. This directly relates to our consideration of the influence of microRNAs on Notch decay rates and ectopic ESN formation in the last two sections.

### Parameter sensitivity analysis

In the study of any model, it is important to determine which parameters have the greatest effects on the system. Our model is a high dimensional, nonlinear model with a large number of equilibria, so one would expect that the sensitivity of the model depends on the region of parameter space where the analysis is performed. Some equilibria will have large basins of attraction and will therefore be very robust to parameter changes, while other equilibria will have smaller basins of attractions and will be more sensitive. For this parameter sensitivity analysis, we examine variations of 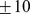
% in each of the parameters for our case where 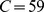 and 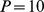, using the other parameter values from Table 1. This equilibrium is associated with a pattern of six neurons with 9 cells between each neuron, and we chose to focus on this equilibrium since this was the mean spacing and neuron count found experimentally and therefore should give us a general idea as to which parameters have a greater effect on our system. We established that the equilibrium for this system was stable and found the eigenvalues.

**Figure 7:**
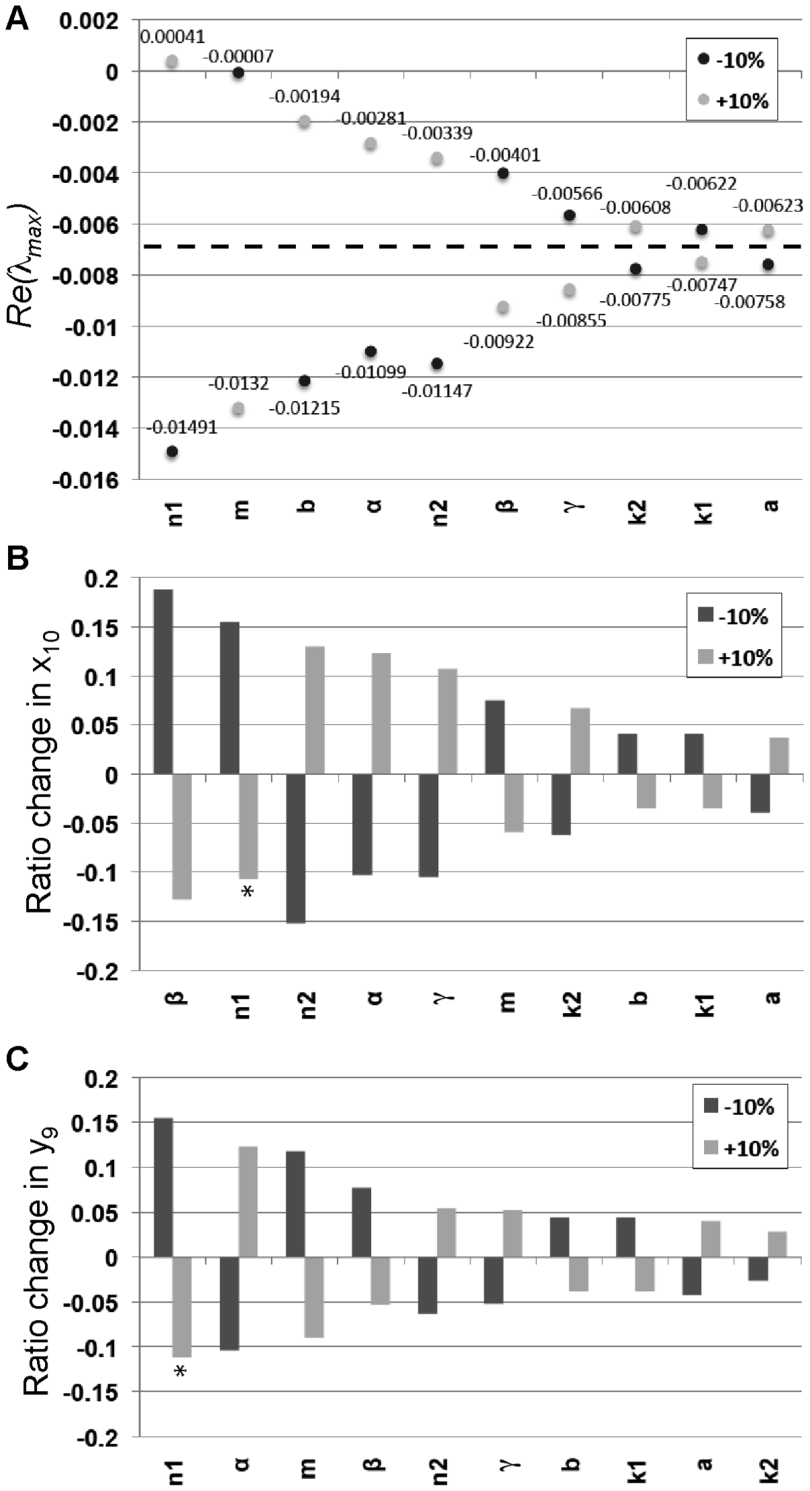
Parameter sensitivity analysis. (A) The values of 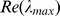 are shown for all the parameters of the model with variations of 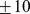
% in each of the parameters. The ordering of the parameters shows which parameters had the largest increase in the eigenvalues for either a 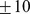
% change with the largest on the left. The dotted line indicates the value of 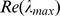 for the original set of parameters used in Table 1. (B) The change in equilibrium value for 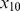 after 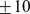
% change in parameter values. The y-axis shows the ratio change of the equilibrium with the new parameter value divided by the original equilibrium value 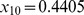. The ordering of the parameters shows which parameters had the largest increase in the magnitude of 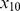 for either a 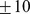
% change with the largest increase on the left. Since the equilibrium is unstable for 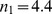, 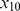 oscillates 17% above and 14% below the equilibrium marked with a 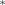. (C) The change in equilibrium value for 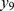 after 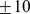
% change in parameter values. The y-axis shows the ratio change of the equilibrium with the new parameter value divided by the original equilibrium value 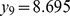. The ordering of the parameters shows which parameters had the largest increase in the magnitude of 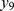 for either a 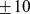
% change with the largest increase on the left. Since the equilibrium is unstable for 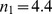, 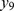 oscillates 13% above and 10% below the equilibrium marked with a 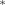.

One measure for the sensitivity is the change in the value of the real part of the maximum eigenvalue. With the base parameters, we found 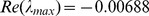. Figure 7A shows that increasing the coefficient of the negative feedback function, 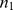, has the greatest effect, and even results in the system going through a Hopf bifurcation. Decreasing the parameter 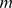 has the next largest effect, which is not too surprising given that its parameter value is close to the Hopf bifurcation for that parameter. As we would expect, the parameters, 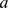, 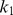 and 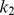 have minimal effect on the eigenvalues, while the other parameters have more varied effects increasing or decreasing the stability. Figure 7A shows the effects of variations of 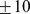
% for all the parameters on the real part of the largest eigenvalue, 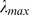.

Our study shows that in the case where 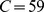 and 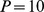, the greatest instability lies in the center between two ESNs. This can be visualized by examining the eigenvector for 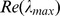. The largest level of 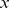 away from the ESNs occurs at 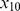, 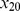,… 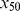 (see Figure 6C). The least stable levels of 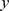 occur in the neighboring cells, such as 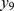 and 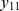 (see Figure 6C). Figure 7B–C provide information on how much a variation of 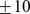
% in a given parameter shifts the equilibrium values at 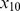 and 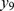, where changes in amplitude are observed to be the largest. When a shift becomes sufficiently large at 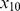 and a threshold is crossed, a new ESN forms in this location, completely changing the equilibrium values for 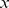 and 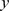. Figure 7 shows that a description of parameter sensitivity for this system depends on the measure that is employed. Clearly, this system is most sensitive to the negative feedback coefficient, 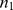. However, the Hill coefficients relate to the degree of cooperativity for binding between Notch and Delta, which are intrinsic properties of the proteins not likely to change over development. As we alluded to before though, decreasing 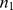 broadens the ESN count and spacing distributions, while increasing 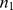 narrows the distributions (Figure S3). We found that a 10% change in 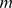 caused a 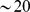
–25% shift in the ESN spacing and count distributions, suggesting that the system is indeed sensitive to this parameter (Figure S3). The least significant parameters are the kinetic constants, 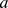, 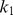, and 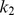. A 10% variation in 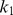 results in a 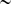
10% change in ESN count and spacing distributions (Figure S3). The robustness of this model to variations in most of the parameters allows reasonable stability for patterning of ESNs, while providing flexibility to produce novel patterns when new adaptations are necessary, such as a need for more or less dense ESNs.

### Incorporation of miR-124 regulation of Notch signaling into the expanded model

We previously showed that the microRNA miR-124 is expressed in the larval midline ESNs of *Ciona intestinalis* [33], [46]. We demonstrated that miR-124 is activated by proneural bHLH genes and negatively regulates Notch signaling by downregulating Notch and all three Hes genes [33], [34] through binding to canonical target sites in the corresponding transcript 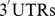 [33]. Mis-expression of miR-124 along the entire epidermal midline increases the number of midline ESNs presumably because of ectopic suppression of Notch signaling [33], although a detailed quantitative analysis was not performed.

Here we generated transgenic embryos using this same miR-124 construct from our previous studies (Epi::miR-124) [33], [46]. We electroporated increasing amounts of the transgene into *Ciona* embryos (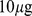, 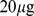, or 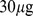; which we denote as Epi::miR-124+10, Epi::miR-124+20, or Epi::miR-124+30, respectively). In each case, we quantified the number and spacing of ESNs at 22 hours post-fertilization, and compared these results to control wild-type 22 hr embryos. We used immunohistochemistry to detect ESN cilia with an anti-acetylated tubulin antibody, and visualized the midlines with either an Ash or Delta fluorescent transgene reporter. We performed each experiment with independent biological replicates, and quantified a total of 17, 19 and 20 embryos for the miR-124+10, miR-124+20 and miR-124+30 experiments, respectively. We only quantified embryos for which we could clearly perform cell counts for both the dorsal and ventral midlines; since miR-124 overexpression produces kinked or twirled phenotypes that make counting difficult, we were not able to quantitate as many embryos as in the wild-type experiment.

**Figure 8:**
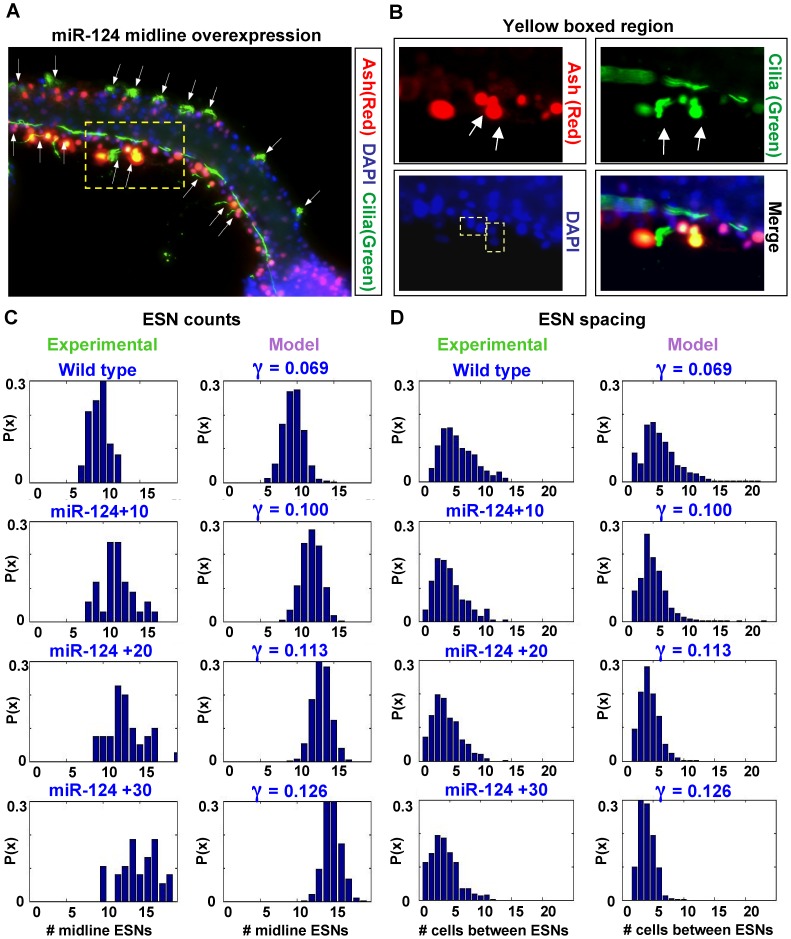
The microRNA miR-124 modulates the Notch decay rate. (A) Representative embryo for miR-124 titration experiments. (B) Magnified region of the embryo in (A) shows adjacent ESNs; DAPI staining shows pairs of small nuclei belonging to the ESN pairs. (C–D) Comparison of the distribution of ESN counts and spacing between miR-124 titration experiments and our model. Based on this data and our previous results [33], [34], we propose that the Notch decay rate, 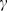, is modulated by miR-124.

Figure 8A–B shows a representative embryo, and the results of our Epi::miR-124 titration experiments are shown in Figure 8C–D. As we increased the amount of the miR-124 transgene electroporated into embryos, the mean number of ESNs per midline increased with a corresponding decrease in the mean ESN spacing. Note that the mean number of midline cells was very similar between the experiments (mean  = 57.7, 57.0, 57.3, 58.9 in wild-type, +10, +20 and +30, respectively), indicating that miR-124 overexpression did not affect the number of midline cell divisions during development (see Figure S1). The largest difference occurred between wild-type and miR-124+10 embryos (difference in mean ESN counts  = 2.44; difference in mean ESN spacing  = −2.44); subsequent increases in miR-124 concentration had a linear effect on the number and spacing of ESNs (average difference in mean ESN counts  = 1.45; average difference in mean ESN spacing  = −1.43). Comparison of ESN count and spacing distributions and the associated minimum/maximum values among the different miR-124 concentrations also showed a shift towards an increasing number of ESNs per midline and decreasing inter-ESN spacing. In particular, the number of zero-spacing cases (*i.e.*, adjacent ESNs) increased as the concentration of miR-124 was increased. A magnified region of the embryo in Figure 8A shows one such case of adjacent ESNs (Figure 8B), which we did not observe in wild-type embryos. This suggests that when expressed at high levels, miR-124 is able to mitigate the effect of lateral inhibition.

Since miR-124 downregulates Notch and Hes by base pairing to their transcript 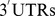 and likely mediating decay at the post-transcriptional level [33], [55]–[58], we proposed that miR-124 regulation of Notch/Hes could be modeled into the Notch decay rate, 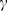. An increase in miR-124 concentration would thus be reflected in our model by an increase in the value for 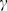. To test this hypothesis, we began with the 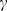 value from our wild-type simulations (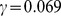) and ran Monte Carlo simulations (M = 1000) continuously increasing the value of 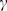 to see if we could match the average number and spacing of high-Delta cells with the number of ESNs in our miR-124 titrations. In agreement with our experiments, we showed in the previous section that continuously increasing 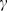 eventually resulted in the formation of new neurons, suggesting that production of extra midline ESNs could be explained by an increase in the Notch decay rate. Indeed, as we continued to increase 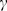, the average number of ESNs continually increased. Eventually, we found values for which both the mean ESN counts and inter-ESN spacing closely matched the observed values in each of the miR-124 titration experiments (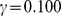 for miR-124+10; 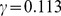 for +20; 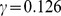 for +30, Figure 8C–D). The marginal increase in 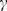 is greatest from wild-type to miR-124+10 embryos, correlating with the high marginal increase of ESN counts between these two samples. Importantly, the distributions of ESN counts and spacings closely fit the experiments, with the model also showing corresponding shifts in the distributions upon increasing gamma values (Figure 8C–D). The variance of the model is smaller than the corresponding miR-124 experiments, even though in the wild-type case the variances were similar between model and experiment. However, overall the model agrees very well with the miR-124 experiments, even more surprising given the fact that we can mimic the experimental ESN patterns with the tuning of just a single parameter. Coupled with extensive biological support [33], [55]–[58], we conclude that our model can accurately incorporate the experimental effect of miR-124 into the Notch decay rate term.

### Evidence that expanded model explains regulation and patterning in sensory cell types across bilaterians

Notch signaling regulates the specification and patterning of sensory cell types not just in *Ciona*, but throughout metazoans (reviewed by [5], [10], [26], [53]). Examples of processes regulated by Notch-Delta signaling include the mechanosensory bristles (macrochaetes and microchaetes) of *D. melanogaster* [30], [31]; the inear ear hair cells of zebrafish [59]–[61], chick [41], [62], and mouse [41], [63], [64]; and the multiciliated cells derived from the respiratory airway epithelium in humans [65], [66]. Interestingly, the sparse patterning in *Ciona* appears also to be found in other animals [7], [30], [31], [35], [37], suggesting that long-range Notch-Delta signaling is also conserved.

Since the inner ear hair cells of vertebrates are likely evolved from the sensory neurons of invertebrates [26], [35], we originally hypothesized that miR-124 regulation of Notch signaling, as we described for *Ciona* [33], should be conserved. However, we found very little published evidence of miR-124 regulating miR-124 outside of ascidians other than miR-124 regulation of Hes1 in the mouse inner ear [67]–[69]. Our own bioinformatic analysis showed that miR-124 rarely targets Notch pathway genes in other organisms [33]. Interestingly though, miR-9 in *Drosophila* appears to regulate Notch signaling in a somewhat analogous fashion [70], suggesting that different organisms deploy different miRNAs to regulate Notch signaling [33]. This would suggest that incorporation of miRNA function into the Notch decay term of our expanded model may be relevant for other systems.

To determine if this might be the case, we first examined the literature to identify sensory neuron-expressed miRNAs in *Drosophila*, zebrafish, mouse, and human. For miR-124, we only found one study in mice where weak miR-124 expression was reported in the vertebrate inner ear [67]. However, many other miRNAs are highly expressed during mouse inner ear development [67], [71], [72]. In other bilaterians, different miRNAs are expressed in these sensory cells, with no obvious conservation of particular miRNA expression (*Drosophila*: [9], [73], [74], zebrafish: [71], human [65], [66]).

**Figure 9:**
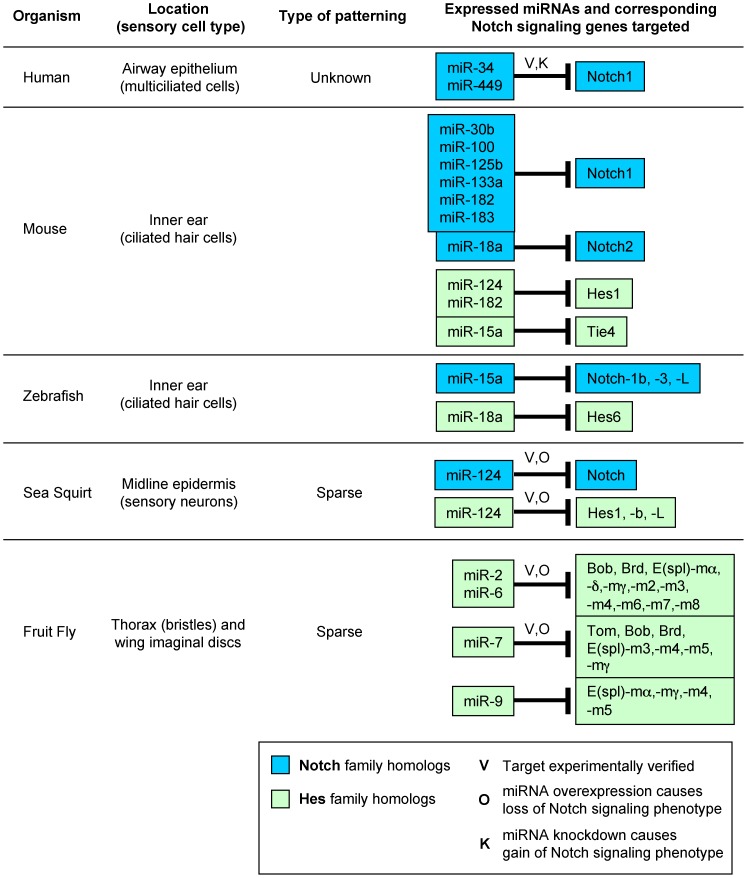
Canonical target sites for miRNAs expressed in sensory cell types throughout bilaterians. Notch signaling regulates the differentiation and patterning of each of the sensory cell types shown. Sensory cell expression for each of the miRNAs listed was shown previously (see text for references). miRNA canonical target sites in the 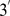
UTRs of Notch and Hes homologs were found using our previously described target prediction algorithm [33]. Some of these targets have been experimentally verified (V) (human: [65]; sea squirt: [33]; fruit fly: [9]). In *Drosophila*, miR-2, miR-6 and miR-7 overexpression (O) have all been previously shown to cause a phenotype indicative of suppressed Notch signaling activity and loss of lateral inhibition, such as increased density or clustering of microchaetes [9]. In human airway epithelial tissue, knockdown (K) of miR-449 has been shown to cause a decreased rate of ciliated cells indicative of Notch signaling gain-of-function [65].

We then bioinformatically searched for canonical target sites of these sensory miRNAs in the 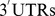 of Notch pathway genes in these animals using a target prediction program we developed previously [33]. Through this, we discovered the presence of predicted target sites in the primary Notch receptor (Notch1) among vertebrates, as well as target sites for other Notch homologs in zebrafish and mouse (Figure 9). In agreement with our hypothesis, we found Notch1 target sites for different sensory miRNAs in each of the different organisms (miR-124 in *Ciona*, miR-15a in zebrafish, miR-30b, −100, −125b, −133a, −182 and 183 in mouse; and miR-34 and miR-449 in human airway epithelium). Among these, miR-34 and miR-449 targeting of Notch in human airway epithelial tissue has been experimentally verified [65]. We did not find any target sites for Notch in *Drosophila*, suggesting that such sensory miRNA regulation of the Notch receptor did not evolve until at least after ecdysozoans.

In agreement with previous reports, we also observed sensory miRNA target sites within many Hes homologs in *Drosophila* [9], [75]. However, whereas in *Drosophila* and *Ciona* almost all of the Hes homologs have target sites, we observed that in vertebrates predicted targeting of Hes is much more restricted (Figure 9). This may be explained by the fact that predicted targeting of the Notch receptor appears to be much more extensive in chordates (Figure 9). Since the Notch receptor is the initial effector of Notch signaling, miRNA-mediated suppression of Notch would relieve the need to target all of the downstream Hes factors. Another possible explanation is that the other Hes factors are not expressed in the sensory cells of vertebrates, and therefore their targeting by sensory miRNAs is not needed. Indeed, among the many Hes homologs in mice, only Hes1 and Hes5 are expressed in inner ear cells, of which Hes1 is the more highly expressed factor [27], [76].

Finally, we note that although in *C. elegans* there is no published evidence of Notch signaling regulating sensory neuron formation, the Notch homolog LIN-12 regulates the formation of some of the adjacent interneurons that relay signals from the sensory neurons [77]. Recent evidence suggests that the miR-51-56 family is ubiquitously expressed among neurons in *C. elegans* [78], and we bioinformatically found a canonical target site for this family of miRNAs in the LIN-12 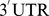. Although in this work we focused on sensory cell types in other organisms, since they are most analogous to the epidermal sensory neurons of *Ciona*, but it would be interesting to explore miRNA regulation of Notch signaling in other cell types.

## Discussion

### Our experimental results in *Ciona* and previous related studies motivate an expanded Notch-Delta model

Previous Notch-Delta models [12], [20]–[24] were based on early Notch signaling studies in *Drosophila*, *Xenopus*, and mouse [2], which suggested a checkerboard expression pattern whereby neighboring cells adopted alternate cell fates. This was supported by evidence in *Drosophila* that cells selected to become neurons activate Notch signaling in neighboring cells thereby preventing these cells from likewise adopting a neuronal fate [5]. This led to the classic Monk model [20], which provided the foundation for later models [12], [21]–[24].

However, more careful analysis has shown that the pattern produced by Notch-Delta signaling in some cases is not checkerboard. For example, the sensory microchaetes of the *Drosophila* thorax are initially formed at every other cell and prevent immediately neighboring cells from adopting a sensory fate via lateral inhibition. However, once the thorax has fully developed, these microchaetes become spaced about 4–5 cells apart. Meanwhile, the larger macrochaetes can be spaced dozens of cells apart [29], [30], [38]. In these cases, it has been suggested that dynamic filopodia extensions may provide a mechanism whereby the Delta ligand can activate Notch signaling in non-neighboring cells [28], [30]. Other examples of experimentally observed non-checkerboard patterning include sparse patterning of bristle cells in other fly species [38]; opposing gradients of Notch versus Delta expression along the apical-basal axis in the developing retina of both mouse and zebrafish [7], [37]; and gradient expression of Notch in the mouse inner ear [41]. These examples suggest the need for updated Notch-Delta models that can reproduce these non-checkerboard patterns. One such model has recently been developed for describing the sensory bristle patterning in *Drosophila* incorporating filopodia extensions [31]. This model requires dynamic lengthening and shortening of filopodia and incorporates data on several variables such as length of filopodia, lifetime of filopodia and sensitivity of Notch signaling to the Delta ligand specific to their experiments. More general models of juxtacrine systems have explored periodic patterning with longer wavelengths, producing sparser patterns also [42]–[44].

Here, we developed an expanded Notch-Delta model that builds upon the minimal equations established first by Monk and later Elowitz and colleagues [12], [20]. Our model incorporates a simple activity gradient that allows for long-range cell communication through juxtacrine (cell-cell) signaling. This is actually a long-range inhibition, mediated by local juxtacrine signaling, using a linear gradient term similar to Fickian diffusive flux. As mentioned earlier, specific examples of Notch-Delta patterning in *Drosophila* [28]–[30] and other fly species [38], as well as in the mouse inner ear [41], mouse retina [37] and zebrafish retina [37] all demonstrate that sparse or gradient neuronal patterns can arise from a field of neurocompetent cells. Unlike the model for *Drosophila* neuronal patterning [31], our model does not require the existence of dynamic filopodia extensions, and actually makes very few assumptions regarding the exact pattern of neurons and the underlying mechanisms responsible for neuronal patterning. Our model can produce a large number of possible equilibrium states and, although here designed for a linear array of cells, is easily adaptable to a planar field of cells. Therefore, we suggest that our model is adaptable and able to reproduce a variety of both sparse and dense spatial patterns, and should be useful for modeling other Notch-Delta systems.

In this study, we applied our model to the patterning of sensory neurons in the peripheral nervous system of *Ciona intestinalis* larvae. In a previous report [33], we found that the array of cells along the *Ciona* midlines are all neurocompetent and can be converted into neurons by inhibiting Notch signaling. However, in wild-type animals only a few of these cells are selected to become ESNs. Specifically, the spatial pattern of ESNs in the larvae of *Ciona intestinalis* is sparse and irregular, with variable ESN spacing ranging from one to thirteen cells between consecutive ESNs in wild-type animals. The large number of non-ESN cells found between one ESN and the next demonstrates the need for an extra term for producing long-range ESN patterns along the midline. This is the motivation for updating the previous Monk and Elowitz models with the addition of a Notch gradient term. For this study, we represent this long-range term as a simple activity gradient, and demonstrate that this is sufficient for explaining the patterning of ESNs in *Ciona*. We note that this is not necessarily a diffusion term, since Notch and Delta are membrane-bound and in most cases do not produce diffusible species. We chose a linear gradient over other possibilities, such as Hill function interactions [44], because it is the simplest and most generic form, and can be applied to a wide variety of biological and physical systems without assuming anything about the underlying mechanisms of long-range communication. One possible mechanism of long-range communication via Notch signaling in *Ciona* may be through the protein 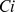-fibrinogen, which is secreted from the tail notochord and is known to interact with Notch in the *Ciona* central nervous system [79]. 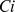-fibrinogen is similar to the fibrinogen-like protein Scabrous, which is involved in producing large-cell bristle spacings in *Drosophila* [28]. We will explore this and other possibilities in future studies and will update our model accordingly.

Finally, we provide a strong mathematical foundation for our model by performing rigorous stability analyses and bifurcation analyses of the key model parameters: the neuron spacing (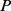), the Notch decay rate (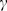), and the slope of the linear gradient (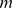). The sensory neurons in *Ciona* derive from bipotent precursor cells along the tail midline, which adopt either an epidermal or neuronal fate [32]–[34]. Our eigenvector/eigenvalue analysis for different spacings between neurons demonstrates that the cells committed to becoming neurons occupy regions of high stability, while epidermal precursor cells more centrally located between consecutive neurons occupy regions of instability. These centrally located cells thus maintain their bipotent character. These cells may have very small basins of attraction for maintaining low levels of Delta and thus, are sensitive to perturbations and small changes in the parameters of the system. As we varied the parameters 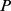, 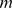, and 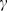, we discovered a threshold phenomenon whereby the system increasingly loses stability to a point where it jumps to a new equilibrium with these central cells becoming neurons. For 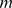 and 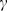, these cells exhibit a hysteresis effect and remain committed to a neuronal fate (*i.e.*, express high levels of Delta), even if the parameters are adjusted back to their original values.

### Motivation for a Notch gradient term

An early study using both *in situ* hybridization and immunostaining demonstrated an apical-to-basal gradient of Notch expression within neuroepithelial precursor cells in the diencephalon, telencephalon, retina and spinal cord during chick development [36]. More recently, apical-to-basal expression gradients of Notch were also found within neuroepithelial cells in the zebrafish retina, where Notch-Delta signaling is active [37], [80]. The nuclei within these neuroepithelial cells are able to migrate along the basal-apical axis, and depending on where these nuclei are within the Notch gradient, after mitosis the daughter cells either remain in their precursor state or differentiate into neurons. Although these studies were examining intra-cellular gradients, this motivated us to consider the possibility that Notch gradients exist between cells along the midline. Intercellular gradients induced by cell-cell signaling relays have been well-established for TGF-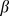 family signaling [44], [81], and although not yet definitely shown to cause gradient patterns, signaling relays also exist in the context of Notch-Delta signaling through Notch activation of secondary relay ligands such as Jagged/Serrate [2], [41].

We found here (Figure S4) and also in previous studies [32]–[34] that Delta expression is restricted to the presumptive ESNs and is not expressed in the other midline cells, therefore a Delta-mediated gradient is not appropriate. Studies of the morphology of *Ciona* sensory neurons found no evidence for dynamic filopodia extensions in the PNS [51], and so the Cohen model is also not appropriate [31]. Conversely, Notch is expressed in all midline cells and, therefore, could mediate long-range communication [6]. Also, from our previous experiments [33], [34], we know that blocking midline Notch signaling using a dominant-negative form of the dowstream effector gene Suppressor-of-Hairless results in ectopic neuron formation along the entire midline. On the other hand, ectopic activation of Notch signaling along the entire midline through mis-expression of Delta causes a reduction in midline neuron formation and large regions without ESNs [34]. Thus, given our experimental knowledge in *Ciona* and knowledge of long-range patterning in other systems, our current hypothesis is that the Notch signal is somehow relayed from ESN-neighboring cells to more distant cells. Therefore, the most reasonable term to add to the original Collier model, given our experimental observations, would be a Notch activity gradient. From a dynamical systems perspective, this is also the simplest form in our model that can produce distal spacing patterns. This Notch gradient may be produced through lateral induction of secondary Notch ligands as in other animals [2], [41]. In *Ciona*, it is known that 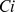-fibrinogen interacts with Notch to regulate neuronal patterning in the central nervous system [79]. Given that a similar Notch ligand, Scabrous, is involved in producing long spacings in the *Drosophila* PNS [28], it is possible that 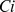-fibrinogen may also act as a Notch ligand in the PNS as well. We will be exploring these and other possibilities in the future. Overall, the linear Notch gradient term provides a simple initial model, which explains the Notch-Delta-mediated patterning of sensory neurons in *Ciona* based on our current biological knowledge of the *Ciona* PNS, and motivates future experiments and updated models.

### Future work and refining our model

The model is a high dimensional system of ordinary differential equations with many equilibria and 11 parameters, including the number of cells in the system. Scaling could be used to eliminate three parameters, but that still leaves 8 parameters. We provided detailed studies for the parameters 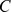, 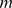, and 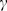, which are significant in *Ciona*, and demonstrated when bifurcations occur, leading to new ESNs forming. In order to examine the stability of ESN patterning, we have conducted some initial bifurcation studies, finding Hopf bifurcations and indications of hysteresis effects through saddle node bifurcations. In the future, more detailed bifurcation studies will be performed to determine the exact type of bifurcation occurring when the miR-124-related parameter 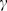 is varied. In addition, we performed a sensitivity analysis for all the parameters about an equilibrium of six high-Delta ESNs with a mean ESN spacing of nine cells to show the relative effects of each parameter as they varied, thus giving a local understanding of the most significant parameters. The model did prove to be quite robust for this equilibrium, producing similar ESN patterns for a range of each of the parameters. We do note that the system was sensitive to small changes in 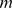. This could suggest that an organism has limited variability in its cell to cell communication, or this could be a potential limitation of our model. More experimental evidence is needed to decide the precise nature of the long-range inhibition, and it is possible that our model will require additional nonlinear juxtacrine signaling functions from lateral induction and/or inhibition. For this current work, we have added a simple linear gradient term and have shown that this is sufficient for producing the long-range patterning of ESNs in *Ciona*.

In order to fit our experimental observations, future work needs to be done on modifying the local interaction terms. Monte Carlo simulations of our model produce too many one-cell spacings compared to what we observe in wild-type larvae. When we adjusted the Notch decay rate parameter, 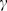, in order to simulate the miR-124 overexpression experiments, the model was not able to produce the zero-spacing adjacent ESNs of miR-124 overexpressing embryos. There are several possibilities for these discrepancies. In the miR-124 experiments, wild-type ESNs endogenously produce miR-124, therefore the actual Notch decay rate is much higher within ESNs compared to the other midline cells. In our model we use a single 'average' 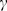 value for all midline cells that does not take this variation in Notch decay rates into account. Indeed, if we increase 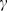 significantly (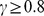), we are able to override lateral inhibition and produce adjacent high-Delta cells. Thus, a more appropriate model may be one that incorporates a spatially-varying 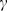, whose form perhaps follows a Gamma distribution. Another possibility is that the level of lateral inhibition (*i.e.*, the strength of the Hill equations) is too strong in our initial model, and that tuning of the Hill coefficients may allow for production of adjacent ESNs more easily. Finally, there may be other yet unidentified local factors that counterbalance the feedback effect between neighboring cells, which we have not accounted for in our model. We will explore each of these possibilities in future studies.

Finally, although our model is motivated by our studies on Notch-Delta signaling in *Ciona*, we emphasize that it can also be applied to many other biological and physical systems. At its core, we have developed a general mathematical model involving two chemical species, 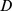 and 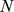, which interact locally as well as over a distance. Local interactions involve a positive and negative feedback governed by Hill functions, which were originally derived by Goodwin [25] to model the reaction kinetics between two biochemical species and for which extensive experimental evidence exists [12], [20], [25]. Distal interactions are governed by a linear activity gradient, which is the simplest and most generic gradient form. Since the specific mode of distal interaction has not yet been determined in *Ciona*, this gradient is appropriate, since we do not assume these are diffusible species and are making no assumptions about the biological mechanism of long-range patterning. The presence of distal interaction greatly expands the number of possible equilibrium states of this system. Finally, as seen from our bifurcation analyses of several parameters, this is a high-dimensional system rich with at least hundreds of possible equilibrium steady states and a variety of interesting dynamics for which we have only begun to explore in this report. Since our model involves only two species and a minimal set of parameters, it is applicable not only to Notch-Delta systems, but is general enough to be applied to analogous biological and physical systems that exhibit both local and distal effects.

## Materials and Methods

### Wild type and miR-124 titration transgenic assays

All of our transgene vectors were cloned in a pSP72 vector backbone (Promega) containing an SV40 Poly(A) site [33], [34], [46], [82]. To visualize expression in the midline, we used two promoter constructs fused in frame with an optimized form of yellow or red fluorescent protein [83]. The first, Ash, contained the conserved *cis*-regulatory and promoter region of the Acete-scute homolog, which showed expression along dorsal and ventral midlines in tailbud embryos [34]. The second construct, Delta, contained a conserved *cis*-regulatory and promoter region as well as the conserved first intron of the Delta2 gene, which is expressed in the *Ciona* PNS [34]. We generated transgenic embryos by electroporating 10-15* µ*g of each construct into fertilized and dechorionated embryos as previously described [46], [83]. Both of these constructs showed midline expression with occasional ectopic expression elsewhere in the epidermis, although the number of expressing midline cells varied from embryo to embryo. This is due to the fact that the genes themselves turn off early in the midline, although the fluorescent proteins have a half-life of 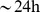 and often remain expressed in the cells. DAPI staining of nuclei and acetylated tubulin antibody staining of cilia was performed as previously described [34]. Images were taken at 20× and 40× magnification with a Zeiss AxioPlan 2e fluorescent microscope equipped with an AxioCam HrM monochromatic camera.

### Detailed explanation of expanded Notch-Delta model

Our expanded Notch-Delta model represented by the ordinary differential equations in (1) begins with a linear array of 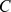 cells. Later we plan to modify this linear array to a dynamic array, which includes cell division. Each cell tracks activity levels of two biochemical species, 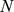 and 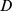. Importantly, note that our model can be generalized to represent the signaling between any two biochemical species, although for this report we focus on Notch-Delta signaling. All the cells in the linear array interact with their nearest neighbors with the exception of the end cells. Here, our model uses average levels of species 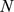 and 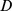 as the missing neighbor for the end cells. The model localizes D inside the cell or expressed on the cell surface to signal only the neighboring cells. It is repressed internally by 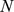 and activates neighboring cells to stimulate production of 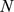. The species 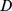 also catalyzes the degradation of 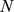 inside the cell. Both species have linear decay terms based on the natural half-lives of 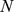 and 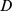. The production of 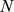 depends on the level of 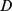 in the neighboring cells. We also include a gradient term for 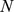 based on the difference in Notch activity between the cells.

The functions and the parameters in the model given by the ODEs in (1) are common in biochemical control models. In the 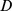 equation, the first function is a standard negative feedback or repression function. The parameters 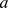 and 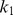 are primarily scaling parameters in the production of 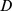. The most significant parameter is 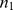, which is the Hill coefficient and reflects the strength of the negative feedback. The higher the value of 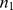, the more effective 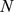 works as a repressor in the production of 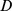. It is well-known that this parameter should significantly affect the stability of the system with larger 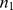 values increasing instability. The parameter 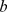 affects the half-life or linear decay of 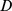. From the indexing in the equation it can be readily seen that production and decay of 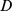 is completely contained in the cell where 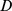 is produced.

The 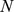 equation is more complicated. The first term represents enhanced *cis*-inhibition of 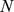 by 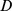 inside the cell. Thus, 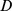 accelerates the degradation of its repressor 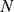 with a scaling parameter 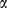. The second term is a non-linear positive feedback or induction function, which has surface molecules of 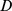 on neighboring cells signaling the production of 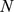. The parameters 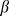 and 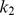 are scaling parameters, while the parameter 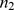 is the Hill coefficient representing the strength of the positive feedback. Again, the higher the value of 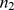, the more switch-like the behavior of this production term for 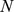 by the levels of 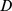 in neighboring cells. The parameter 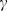 is the linear decay rate for 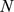. The last term is a gradient term for communication of 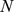 between neighboring cells. This is a standard linear gradient term for flux of 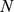 with a rate of 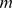 between cells with differing activity levels of 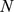. The value of 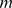 will affect the range of communication or signaling of 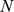 with higher values of 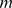 corresponding to longer range signaling.

### Stability analyses and Monte Carlo simulations

The system (1) was coded and simulated in Matlab (R2008b, revision 20) using the 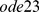 solver. Stability and bifurcation analyses on the parameters 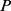, 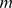 and 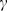, as well as all Monte Carlo simulations were performed using custom Matlab scripts.

### Linearization of the system

For stability analysis we need to linearize the system (1). We let 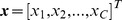 and 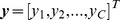 and write the system (1) as follows: 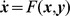


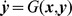



We assume an equilibrium solution 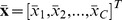 and 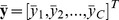, then we define the perturbed variables from the equilibrium as 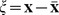 and 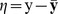. The linearized version is written 
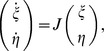
where 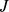 is the Jacobian matrix with 
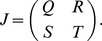



The 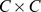 submatrices 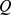, 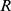, 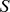, and 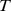 are created with their 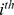 row and 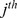 column satisfying: 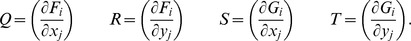



Submatrices 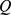 and 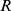 are relatively simple with only diagonal form. All diagonal elements for 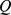 are 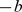. Since 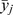 is the 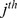 equilibrium value for 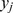, the diagonal element for 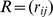 satisfies: 
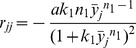



The diagonal form of the matrices 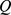 and 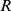 reflect that the substance 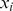 is confined to the 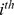 cell. The diagonal elements in 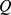 reflect the linear decay of 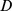 in the model. The diagonal elements in 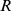 reflect the production of 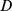, which is repressed by 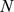.

Since the 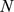 variable of the model is produced and communicated based on the neighboring cells, the submatrices 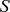 and 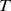 are predominantly tridiagonal. The values 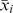 and 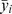 are the 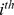 equilibrium values for 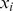 and 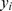. For the submatrix 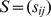, the diagonal elements are predominantly 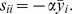



This term reflects the enhanced degradation of 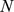 by 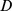 in the cell. The subdiagonal and superdiagonal elements come from the production term with most satisfying 
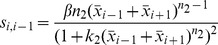



This reflects the enhanced production of 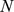 by 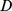 in the neighboring cells.

For this version of the model we chose to make the boundaries substitute the average activity level for the end levels. This leads to small contributions in the 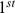 and 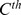 rows of 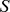. In addition to the terms listed above for 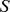 we add the terms: 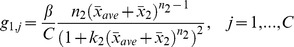
and 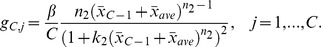



For the submatrix 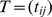, the diagonal elements are predominantly 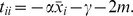



This term reflects the enhanced degradation of 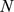 by 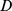 in the cell, the linear decay of 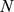, and 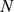 part of the gradient term. The subdiagonal and superdiagonal elements come from the other terms of the gradient 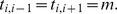



Since the boundaries use the average activity level for the end levels, we obtain small contributions in the 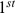 and 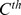 rows of 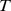. In addition to the terms listed above for 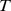 we add the terms: 
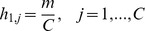
and 
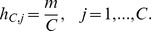



### Equilibria

We noted that system (1) has many equilibria. For the Monte Carlo simulations we began the simulations with random low values (both 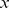 and 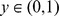) and allowed long simulation times for solutions to settle into a stable pattern of ESNs, which is determined by the ESNs, where 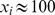. Most of these equilibria did not have their eigenvalues tested, so several of the stable patterns of ESNs, undoubtedly had eigenvalues with positive real parts, making system (1) unstable and leaving some cells to have low amplitude oscillations. (See Movie S2.) These oscillating cells could be considered bipotent, but the local environment remains sub-threshold, so they fail to convert to ESNs.

For the bifurcation studies in spacing, 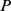, the gradient parameter, 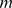, and the Notch decay parameter, 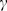, initial conditions were provided that favored a particular pattern. For some patterns with certain parameter values, the basins of attraction for the particular pattern were very large, and simulations easily settled to the desired pattern with initial conditions only roughly exhibiting the planned pattern. Nearer bifurcation points, the initial conditions required using equilibria from nearby (stabler) parameters. The equilibria used for stability analysis were found by a long time simulation "near'' a particular equilibrium. Subsequently, this simulated equilibrium had Newton's method with the Jacobian shown above applied to system (1) with the derivatives set to zero. The equilibrium results from the Newton's method were used for the local analysis described above to find eigenvalues and eigenvectors.

## Supporting Information

Figure S1 **Cell counts in the dorsal and ventral midlines.** The average number of midline cells, ESNs and number of cells separating consecutive ESNs (spacing) were counted for wild-type (wt) and miR-124 overexpression embryos (+10, +20, +30). Significant differences between dorsal and ventral midlines are marked with an asterisk (p<0.05, *t*-test with multiple testing correction). Errors bars indicate SD. In the text, we consider statistical averages per midline without distinction between dorsal and ventral midlines, since overall across all experiments there are no substantial differences between dorsal and ventral counts.
(EPS)

Figure S2 **Large ESN spacings are flanked on at least one side by a pair of closely spaced ESNs.** (A) Representative image of a large (11-cell) spacing flanked on the anterior side by two neurons closely spaced. (B) Representative simulation run of our model showing the same phenomenon.
(EPS)

Figure S3
**ESN counts and spacing distributions for changes in the parameters** 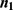 **,** 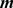 **and** 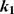 **. **(A–D) Monte Carlo simulations were performed when increasing or decreasing 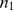 and 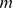, the more sensitive parameters identified in our parameter sensitivity analysis. The resultant distributions are shown for ESN counts (left) and spacing (right) for increasing or decreasing 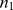 from the original value of 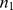 = 4 with 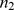 held constant (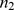 = 3) (A–B) and for increasing or decreasing 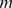 by 10% from the original value of 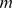 = 0.10 (C–D). (E) Resultant distributions from running Monte Carlo simulations after increasing the value of 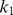 by 10%. The mean 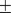 standard deviation of the distributions are as follows: (A) ESN count  = 8.07 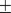 2.25, spacing  = 5.30 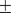 4.51; (B) ESN count  = 7.46 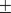 1.05, spacing  = 5.83 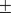 2.40; (C) ESN count  = 10.83 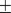 1.34, spacing  = 3.94 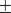 1.86; (D) ESN count  = 6.93 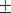 1.30, spacing  = 6.27 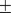 3.35; (E) ESN count  = 7.56 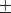 1.46, spacing  = 5.78 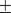 3.34. For the original parameters (Table 1, Fig 4), the Monte Carlo simuations produced average ESN count  = 8.48 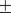 1.37, average spacing  = 5.12 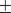 2.84.(PDF)

Figure S4 **Delta expression in the tail midlines detected using** ***in situ*** **hybridization.** Delta shows specific expression in the presumptive ESNs along both the ventral and dorsal tail midlines.
(PDF)

File S1 **MATLAB code for simulating the expanded Notch-Delta model.**
(TXT)

File S2 **MATLAB code for running Monte Carlo simulations.**
(TXT)

Movie S1 **Simulation run of expanded model leading to a stable equilibrium.** The simulation begins at random low initial conditions. Because of the fast dynamics of the initial patterning, the first seconds are simulated at 5 time units per second, while the remainder of the movie is simulated at 50 time units per second.
(AVI)

Movie S2 **Simulation run of expanded model leading to stable oscillations.** The simulation begins at random low initial conditions. Because of the fast dynamics of the initial patterning, the first seconds are simulated at 5 time units per second, while the remainder of the movie is simulated at 50 time units per second.
(AVI)
