# Supplementary figures and images for: An Expanded Notch-Delta Model Exhibiting Long-Range Patterning and Incorporating MicroRNA Regulation
Source: PLoS Comput Biol. 2014 Jun 19;10(6):e1003655. doi: 10.1371/journal.pcbi.1003655 (PMC4063677; doi:10.1371/journal.pcbi.1003655)

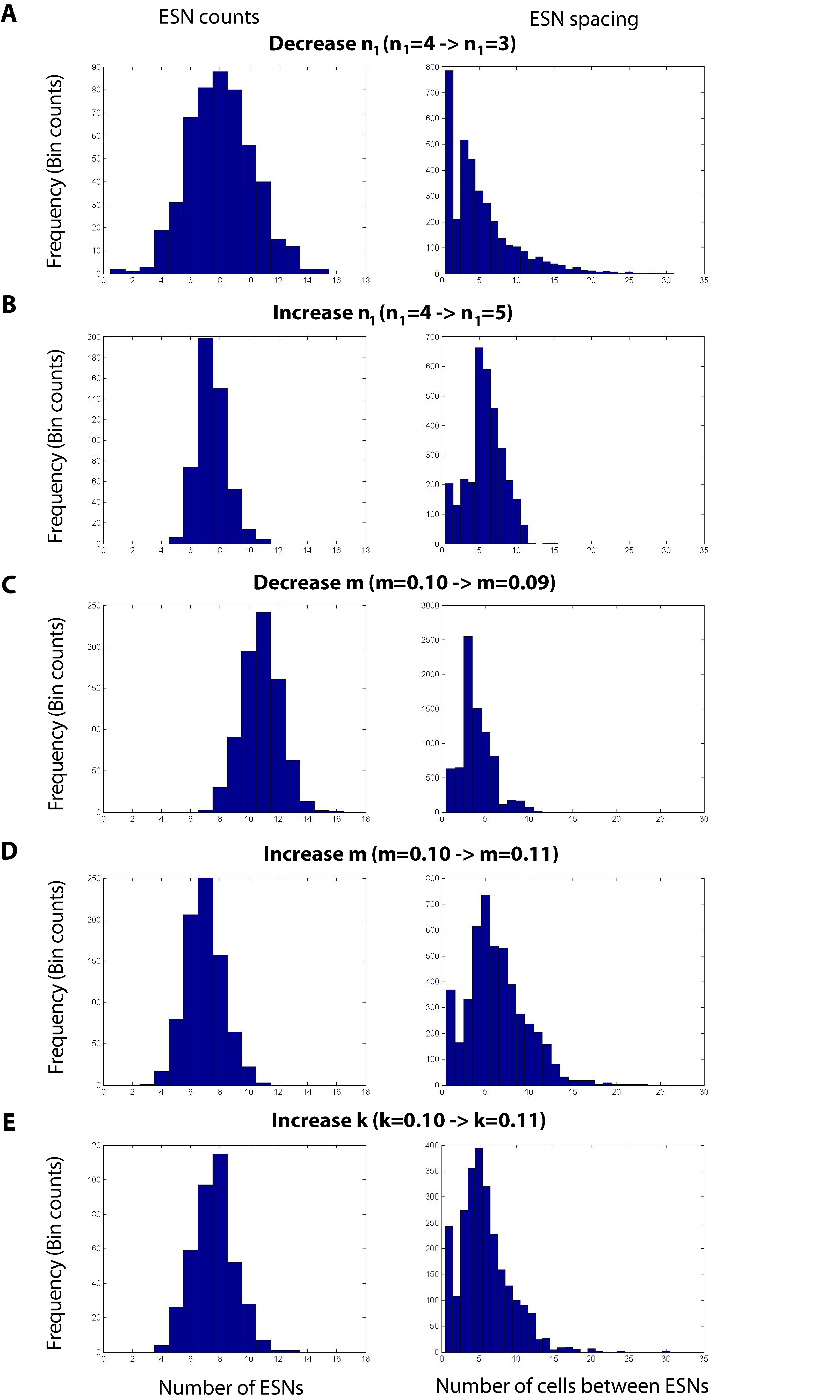

Supplement: Figure S3 — ESN counts and spacing distributions for changes in the parameters , and . (A–D) Monte Carlo simulations were performed when increasing or decreasing and , the more sensitive parameters identified in our parameter sensitivity analysis. The resultant distributions are shown for ESN counts (left) and spacing (right) for increasing or decreasing from the original value of = 4 with held constant ( = 3) (A–B) and for increasing or decreasing by 10% from the original value of = 0.10 (C–D). (E) Resultant distributions from running Monte Carlo simulations after increasing the value of by 10%. The mean standard deviation of the distributions are as follows: (A) ESN count = 8.07 2.25, spacing = 5.30 4.51; (B) ESN count = 7.46 1.05, spacing = 5.83 2.40; (C) ESN count = 10.83 1.34, spacing = 3.94 1.86; (D) ESN count = 6.93 1.30, spacing = 6.27 3.35; (E) ESN count = 7.56 1.46, spacing = 5.78 3.34. For the original parameters (Table 1, Fig 4), the Monte Carlo simuations produced average ESN count = 8.48 1.37, average spacing = 5.12 2.84. (PDF) [file pcbi.1003655.s003.pdf]

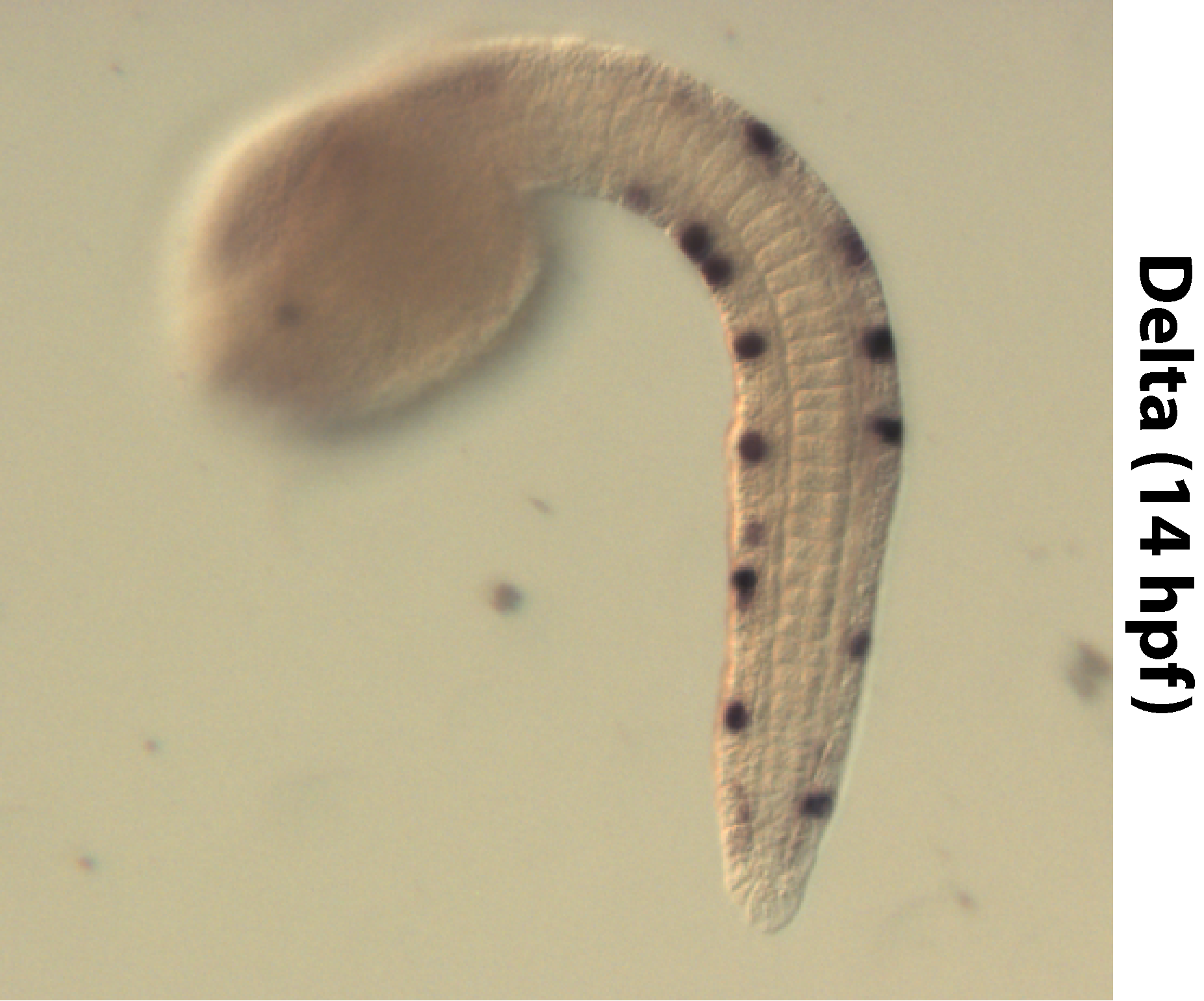

Supplement: Figure S4 — Delta expression in the tail midlines detected using in situ hybridization. Delta shows specific expression in the presumptive ESNs along both the ventral and dorsal tail midlines. (PDF) [file pcbi.1003655.s004.pdf]
